# The highly divergent *Jekyll* genes, required for sexual reproduction, are lineage specific for the related grass tribes Triticeae and Bromeae

**DOI:** 10.1111/tpj.14363

**Published:** 2019-05-25

**Authors:** Volodymyr Radchuk, Rajiv Sharma, Elena Potokina, Ruslana Radchuk, Diana Weier, Eberhard Munz, Miriam Schreiber, Martin Mascher, Nils Stein, Thomas Wicker, Benjamin Kilian, Ljudmilla Borisjuk

**Affiliations:** ^1^ Leibniz‐Institute of Plant Genetics and Crop Plant Research (IPK) 06466 Gatersleben Germany; ^2^ Vavilov Institute of Plant Genetic Resources (VIR) St. Petersburg 190000 Russian Federation; ^3^ Department of Experimental Physics 5 University of Würzburg Würzburg Germany; ^4^ The James Hutton Institute Invergowrie, Dundee DD2 5DA UK; ^5^ Department of Plant and Microbial Biology University of Zürich Zürich Switzerland; ^6^ Present address: Division of Plant Sciences School of Life Sciences University of Dundee The James Hutton Institute Invergowrie, Dundee DD2 5DA UK; ^7^ Present address: Global Crop Diversity Trust 53113 Bonn Germany

**Keywords:** gene alleles, lineage‐specific genes, Triticeae, *Hordeum vulgare*, *Triticum aestivum*, gene family evolution, plant reproduction

## Abstract

Phylogenetically related groups of species contain lineage‐specific genes that exhibit no sequence similarity to any genes outside the lineage. We describe here that the *Jekyll* gene, required for sexual reproduction, exists in two much diverged allelic variants, *Jek1* and *Jek3*. Despite low similarity, the Jek1 and Jek3 proteins share identical signal peptides, conserved cysteine positions and direct repeats. The Jek1/Jek3 sequences are located at the same chromosomal locus and inherited in a monogenic Mendelian fashion. *Jek3* has a similar expression as *Jek1* and complements the *Jek1* function in *Jek1*‐deficient plants. *Jek1* and *Jek3* allelic variants were almost equally distributed in a collection of 485 wild and domesticated barley accessions. All domesticated barleys harboring the *Jek1* allele belong to single haplotype J1‐H1 indicating a genetic bottleneck during domestication. Domesticated barleys harboring the *Jek3* allele consisted of three haplotypes. *Jekyll*‐like sequences were found only in species of the closely related tribes Bromeae and Triticeae but not in other Poaceae. Non‐invasive magnetic resonance imaging revealed intrinsic grain structure in Triticeae and Bromeae, associated with the *Jekyll* function. The emergence of *Jekyll* suggests its role in the separation of the Bromeae and Triticeae lineages within the Poaceae and identifies the *Jekyll* genes as lineage‐specific.

## Significance Statement

The Jekyll gene, required for sexual reproduction, exists in two much diverged allelic variants that share little similarity in sequences but have common functions of the encoded proteins. Jekyll was found only in species of the related tribes Bromeae and Triticeae suggesting its role in tribe separation within the Poaceae. The emergence of Jekyll coincided with innovations in grain structure of the Bromeae and Triticeae lineages and identifies the Jekyll genes as lineage specific.

## Introduction

The incredible morphological diversity observed between individuals of the same and across species is caused mainly by high levels of genetic diversity. Many protein‐encoding gene sequences are strongly conserved between phyla (Douzery *et al*., 2004). These genes tend to display only a limited degree of nucleotide polymorphism, although some variants can have phenotypic consequences. By contrast, other genes are lineage specific: they exist only within a species or a group of closely related species (Fischer and Eisenberg, 1999). This latter class of gene frequently makes a significant contribution to the evolution of lineage‐specific phenotypes (Kaessmann, 2010). The lineage‐specific genes are typically relatively short in length, harbor small numbers (if any) of introns and have evolved rapidly (Toll‐Riera *et al*., 2009). Prominent among such genes are those that mediate sexual reproduction, therefore facilitating reproductive isolation and therefore speciation (Swanson and Vacquier, 2002). The advent of reproductive isolation represents one essential event in the formation of new lineages in the course of evolution.

Among the world's agricultural crops, the tribe Triticeae of the grass family Poaceae has one of the most intriguing genetic and agricultural histories. The tribe houses approximately 360 species dispersed among 20–30 genera (Barkworth and von Bothmer, 2009; Bernhardt *et al*., 2017). The important temperate cereal crops bread wheat (*Triticum aestivum*), hard wheat (*T. durum*), barley (*Hordeum vulgare*) and rye (*Secale cereale*), together with a number of minor crops, are members of the tribe Triticeae (Zohary *et al*., 2012) that are thought to have diverged from the Bromeae around 32–39 million years ago. While the speciation events leading to the formation of the wild ancestors of barley, rye and wheat date to around 8–9 million years ago (Bossolini *et al*., 2007; Brassac and Blattner, 2015; Bernhardt *et al*., 2017). The Near East ‘Fertile Crescent’ has been recognized as one of the primary sites of crop domestication and also as the center of origin and primary diversity of the Triticeae crops (Harlan, 1971; Kilian *et al*., 2009; Zohary *et al*., 2012).

These crops are grown primarily for their grain, a structure composed of three distinct components: the diploid maternal tissue (testa, pericarp and nucellus), the diploid embryo and the triploid endosperm. The endosperm is the primary storage organ and accumulates starch and protein. The endosperm size directly correlates with the size of the grain largely determining grain yield (Gegas *et al*., 2010). Assimilates from the mother plant reach the endosperm predominantly via the maternal nucellar projection and the filial endosperm transfer cells, which are situated in the crease of the grain (Melkus *et al*., 2011). The grains formed by Panicoideae species such as sorghum (*Sorghum bicolor*) and maize (*Zea mays*) are structured differently: they develop a specialized pedicel region in which the basal endosperm transfer layer is responsible for the traffic of assimilate into the filling grain (Wang *et al*., 2012; Sosso *et al*., 2015). In rice (*Oryza sativa*, tribe Oryzeae), at least two pathways are involved in the transport of nutrients to the endosperm: one operates via the nucellar projection and endosperm transfer cells, and the other via the nucellar epidermis (Oparka and Gates, 1981). These differences in the mechanics of grain filling reflect heterogeneity in grain morphology and are under the control of distinct genes (Radchuk and Borisjuk, 2014).

The species‐specific *Jekyll* gene is required for successful sexual reproduction in barley: in its down‐regulation, plants exhibit a substantial loss in fertility and grain weight (Radchuk *et al*., 2006, 2012). The expression of *Jekyll* in the tapetum of the developing anther is important for pollen maturation and anther dehiscence, and the Jekyll protein is deposited at the surface of pollen grains (Radchuk *et al*., 2012). In the developing grains, Jekyll is required to ensure terminal cell differentiation in the nucellar grain tissues and for the direction of assimilate to the endosperm during grain filling (Radchuk *et al*., 2006; Melkus *et al*., 2011). Here it is shown that the gene *Jekyll* is present in two very divergent allelic variants, while a comparative analysis has established that the timing of its appearance in the evolutionary history of the Triticeae and Bromeae coincided with innovations in grain structure.

## Results

### Genomes of diverse barley cultivars contain different Jekyll sequences

**Figure 1:**
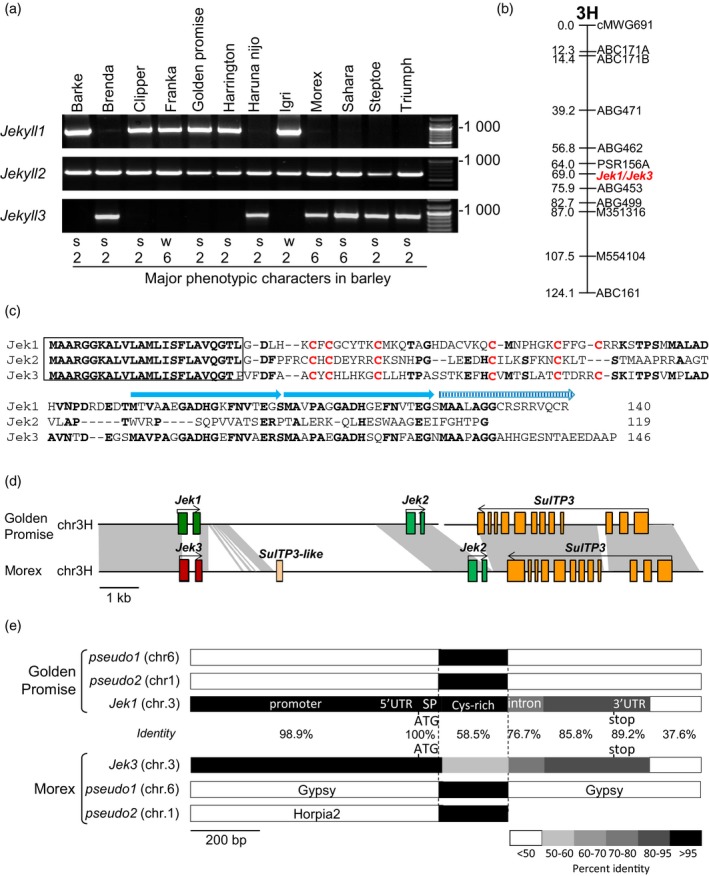
The barley *Jekyll* genes. (a) PCR assays identifying the presence of *Jek1*,* Jek2* and *Jek3*. s: spring type, w: winter type, 2: two row spike, 6: six row spike. (b) *Jek1* and *Jek3* map to the same site on the short arm of chromosome 3H, as deduced from segregation analysis of a mapping population cv. Harrington (contains *Jek1*) × cv. Morex (*Jek3*). (c) The deduced Jek1, Jek2 and Jek3 polypeptides. Residues conserved in at least two of the sequences are shown in bold, signal peptides are boxed, the conserved cysteines are shown in red and the C‐terminal direct repeats in Jek1 and Jek3 are shown by blue arrows. (d) The *Jek1*/*Jek3* locus structure in cv. Golden Promise and cv. Morex. (e) Comparison of genomic regions containing *Jek1* in cv. Golden Promise or *Jek3* in cv. Morex on chromosome 3H and *Jek*‐like fragments on chromosomes 6 and 2 of each cultivar. The scale shown below indicates % identity. ATG, translation start codon; SP, predicted signal peptide; stop, translation stop; UTR, untranslated region.

Although the expression of *Jekyll* has been identified as being essential for sexual reproduction in barley (Radchuk *et al*., 2006, 2012), the further analysis (Figure 1a) in diverse barley germplasm manifested that some perfectly fertile accessions do not harbor *Jekyll* [assigned in the following as *Jekyll1* (*Jek1*)] indicating the presence of other sequences with similar function. A search of a barley EST database (Zhang *et al*., 2004), using the *Jek1* sequence as the query, identified two additional similar sequences, here designated as *Jek2* and *Jek3*. A PCR analysis of 12 barley cultivars differing in major morphological features revealed that whenever *Jek1* was present, *Jek3* was absent, and *vice versa;* meanwhile, the *Jek2* primer pair amplified a fragment from every analyzed accession (Figure 1a). A BLAST scan of the EST content of cv. Barke showed that *Jek1* transcript was readily recovered, but *Jek3* was not.

The *Jek2* and *Jek3* cDNAs were cloned from a library derived from developing seeds of cv. Morex and re‐sequenced. The *Jek1* and *Jek3* nucleotide sequences were 77.1% identical, whereas *Jek1* and *Jek*2 shared 68.6% identity, and *Jek2* and *Jek3* shared 64.6% identity to each other. The predicted amino acid sequences differed significantly from each other resulting in only 51.1% identity between Jek1 and Jek3 and only 26.7% identity between Jek2 and Jek3, or 25.8% identity between Jek1 and Jek2 (Figure 1c). However, all three proteins shared the same putative signal peptide at their N‐terminus and six conserved cysteine residues in their central portion. At their C‐terminus, Jek1 harbored two and Jek3 three almost perfect direct repeats, while Jek2 harbored none (Figure 1c). As for *Jek1* (Radchuk *et al*., 2006), neither the cDNA nor the protein sequences of both *Jek2* and *Jek3* genes shared any significant similarity to any sequence currently in the public domain.

In line with the PCR results, the *Jek1* sequence was found to be present in the draft whole genome sequence of both cultivars. Barke (International Barley Genome Sequencing Consortium, 2012) and Golden Promise, while cv. Morex harbored only *Jek3* (gene ID number: HORVU3Hr1G068160.1; Mascher *et al*., 2017). Both sequences were allocated to chromosome 3H, and both coding sequences were interrupted by a single intron (Figure 1b,d). A comparison of the cv. Golden Promise genomic sequence surrounding *Jek1* with the cv. Morex region surrounding *Jek3* showed that the overall level of nucleotide identity was 98.9% in the 18 400 base pairs (bp) lying 5′ upstream of the gene ATG start codon, 100% in their 5′ UTRs, 100% with respect to the sequences encoding signal peptides and 89.2% in their 3′ UTRs. In contrast, the central portions of the coding regions shared only 58.5% identity (Figure 1e) and were even more divergent than the *Jek1* and *Jek3* intron sequences (76.7% identity). The *Jek2* sequence (HORVU3Hr1G068150.1), almost identical in both cv. Golden Promise and cv. Morex (Figure S1), was also located on chromosome 3H at a site in close proximity to both the *Jek1* locus in cv. Golden Promise (at distance of 4.8 kb) and the *Jek3* locus in cv. Morex (6.3 kb) (Figure 1d). The sequences between *Jek1*/*Jek3* and *Jek2* from cv. Golden Promise and cv. Morex, correspondingly, showed only moderate sequence similarity (63.4%). In both cultivars, a gene encoding a sulfate transporter 3 (*SulTP3*; gene ID HORVU3Hr1G068140.3) was at the closest position to the *Jek* locus (Figure 1d).

Two further cv. Morex sequences exhibiting a degree of homology to *Jek1* were found on chromosomes 6H (physical position 20 756 556–20 756 769 bp) and 1H (physical position 14 743 811–14 743 978 bp): the matching sequence in both cases was similar to a 203‐bp stretch carrying a cysteine‐rich part of the Jek1 protein (Figure 1e). The contiguous sequences at both genomic sites exhibited no similarity to either *Jek1* or any other known genes. As they lacked any long reading frames, the sequences were considered to represent fragmented genes or pseudogenes. This assumption was supported by the fact that the Morex chromosome 6H copy was embedded within a *Gypsy* transposon, while the one on chromosome 1 was flanked by a *Hordeum horpia2* transposon; both the cv. Golden Promise chromosome 6H copy (contig flattened_line_455245) and the chromosome 1H copy (cn433514) were identical to their cv. Morex counterparts (Figure 1e).

We hypothesized that *Jek1* and *Jek3* represent highly diverged alleles of the same gene. The *Jek2* is highly conserved, present in both cv. Morex and cv. Barke and is an additional member of the *Jek* family. Limited similarity in the intergenic regions, flanked by *Jek1*/*Jek3* and *Jek2* genes, points to rapid evolutionary changes at this locus.

### Evidence supporting Jek1/Jek3 allelism

To test whether *Jek1* and *Jek3* are allelic, reciprocal crosses between cv. Barke (*Jek1*) and cv. Morex (*Jek3*) were performed (Figure S2). As expected, all F_1_ plants carried both sequences (five hybrid plants were analyzed for each cv. Morex × cv. Barke and vice versa crossing combinations), which further segregated into the F_2_ generation in the 1:2:1 ratio (with χ^2^ = 0.24, *P *=* *0.89 for 100 F_2_ individual plants of the cv. Morex × cv. Barke cross and χ^2^ = 0.85, *P *=* *0.66 for 120 F_2_ individuals of the cv. Barke × cv. Morex cross). Furthermore, in the cv. Harrington (*Jek1*) × cv. Morex (*Jek3*) double haploid (DH) segregating population (http://wheat.pw.usda.gov/ggpages/HxM/) of 140 lines, each of these carried either the *Jek1* or the *Jek3* fragment but never both (Figure S3b). The *Jek1* and *Jek3* sequences were mapped to the same position of 69 cm of chromosome 3H with a highly significant LOD score of 22.0 (Figure 1b). Only the *Jek3* sequence was detected in parents of the cv. Steptoe × cv. Morex DH population consisting of 105 lines (Figure 1a). However, two SNPs in their *Jek3* fragments allowed the creation of a cleavage‐amplified polymorphic sequence marker (Figure S3d). Using this marker, *Jek3* was again mapped on chromosome 3H (Figure S2c). Similar results were obtained with the cv. Barke × cv. Morex mapping population (Close *et al*., 2009) consisting of 92 lines. *Jek1* and *Jek3* were found to be segregating as allelic variants allowing gene mapping at chromosome 3H, surrounded by Illumina SNP markers 1_1391 (70.9 cm) and 2_1305 (71.8 cm).

Based on the Harrington ×* *Morex population, the *Jek1*/*Jek3* locus resided in the confidence interval associated with a quantitative trait locus (QTL) for grain yield in three out of nine environments (Figure S3a), explaining 10–15% of the variation for this trait (Table S1). Similarly, in the Steptoe × Morex population, the *Jek3* locus overlapped the position of a QTL underlying grain yield in six out of 16 environments (Figure S3b), explaining 20% of the variation for this trait (Table S1). Furthermore, the *Jek1*/*Jek3* locus was associated with grains per ear (−log_10_ *P *= 3.79) in a worldwide spring barley landrace collection (Pasam *et al*., 2012).

### Jek1 and Jek3 are functionally similar despite their sequence divergence

**Figure 2:**
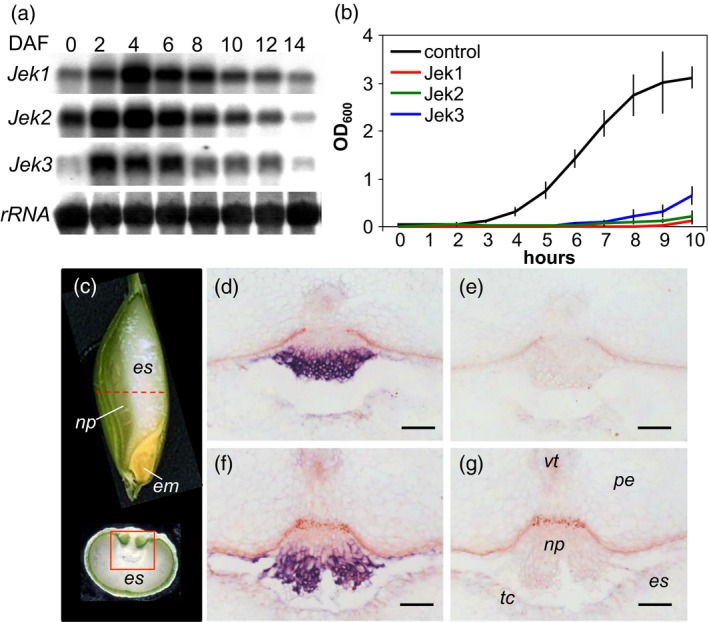
Temporal and spatial expression patterns of *Jek* genes and the cytotoxic nature of their proteins. (a) Northern blot analysis of *Jek1*,* Jek2* and *Jek3* gene expression in the developing barley grains of cv. Barke (*Jek1*) and cv. Morex (*Jek2* and *Jek3*). The loading control used a 25S rDNA probe. (b) The influence of Jek1, Jek2 and Jek3 recombinant proteins on the growth of *E. coli* cells. Values are means (±SD) of five replicates. (c) The barley grain at the mid developmental stage. The red dashed line indicates the plane of the cross‐sections used for *in situ* hybridization sample preparation, and the red rectangle indicates the region shown in (d–g). (d–g) *In situ* hybridization with antisense *Jek3* as probe shows that *Jek3* is strongly transcribed in the nucellar projection at (d) six and (f) 8 days after flowering; (e–g) negative control experiments for, respectively, (d) and (f), using sense *Jek3* as probe. Bar: 100 μm. em, embryo; es, endosperm; DAF, days after flowering; OD_600_, optical density at 600 nm; np, nucellar projection; pe, pericarp; tc, endosperm transfer cell; vt, vascular tissue.

Over the course of grain development in cv. Morex, the abundance of *Jek2* and *Jek3* transcripts was highest approximately 4 days after flowering (Figure 2a), a time that coincided with the peak transcription of *Jek1* in cv. Barke (Radchuk *et al*., 2006). Similarly to *Jek1* in cv. Barke, *Jek3* transcripts in cv. Morex grains were localized in the nucellar projection (Figure 2c–g) as analyzed by *in situ* hybridization. Analysis of *Jek2* expression by qRT‐PCR in microdissected seed tissues (Tran *et al*., 2014) detected the transcripts predominantly in the nucellar tissues, although the abundance of *Jek2* transcripts was one order of magnitude lower than that of *Jek1* (Figure S4). Translation of Jek1 in *E. coli* efficiently inhibited bacterial growth (Radchuk *et al*., 2006). *E. coli* cells, overexpressing Jek2 or Jek3, showed decelerated growth of transgenic versus wild strains (Figure 2b) elsewhere. Therefore, both Jek2 and Jek3 possess cytotoxic properties, similar to Jek1.

**Figure 3:**
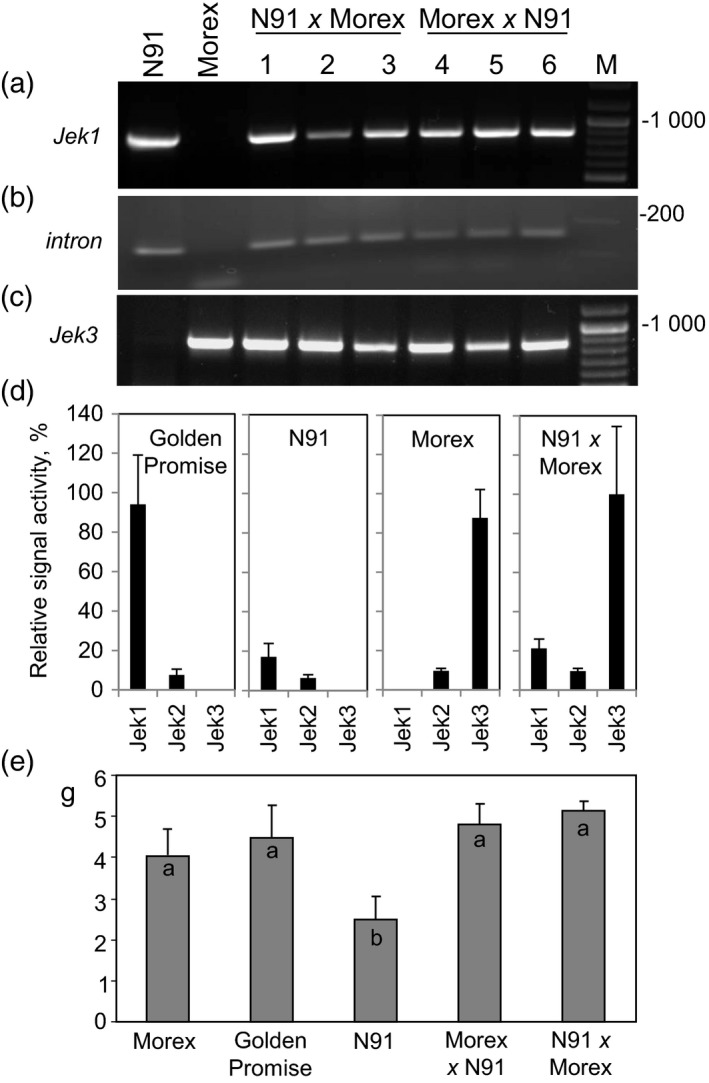
The complementation of *Jek1* by *Jek3*. (a) – (c) Reciprocal hybrids between cv. Morex (*Jek3*) and line N91 (*Jek1* knock‐down line) carry *Jek1* (a), the intron from the RNAi construct used to knock down *Jek1* (b) and *Jek3* (c). (d) Expression of *Jek1*,* Jek2* and *Jek3* in grains of cv. Golden Promise, line N91, cv. Morex and N91 × cv. Morex hybrid, correspondingly. (e) The weight of 100 grains of cv. Morex, cv. Golden Promise, line N91 and the reciprocal cv. Morex × line N91 F1 hybrids. Each data is the mean (±SD; *n = *7); different letters associated with a column indicate statistically significant (*P* < 0.001) differences at upon pair‐wise comparisons in a logistic regression.

To determine whether Jek3 can functionally complement Jek1 *in planta*, crosses were made between cv. Morex (*Jek3*) and line N91. In grains of the line N91, *Jek1* expression had been knocked down via RNAi by approximately 80% compared with untransformed cv. Golden Promise grains, whereas the expression of *Jek2* was not affected (Figure 3d). Line N91 produced fewer grains with significantly lower grain weight due to down‐regulated *Jek1* expression (Radchuk *et al*., 2006, 2012). While cv. Golden Promise (and consequently N91) is a dwarf variety producing two‐rowed spikes, cv. Morex is tall and a six‐rowed variety. The cv. Morex × N91 hybrid plants were tall and produced two‐rowed spikes because of the dominant character of these traits. Molecular analysis of these plants confirmed that they carried both *Jek1* and *Jek3* sequences (Figure 3a,c), as well as the *Jek1*‐RNAi sequence downregulating *Jek1* expression (Figure 3b). *Jek3* expression in the hybrid grains was not affected (Figure 3d) because the only variable part of the *Jek1* sequence was used to prepare the RNAi construct to repress *Jek1* expression (Radchuk *et al*., 2006). The hybrids were characterized by a restored seed set and produced grains of similar weight to those of wild type cv. Morex and cv. Golden Promise plants (Figure 3e), confirming that the phenotype generated by knocking down *Jek1* can be rescued by the expression of *Jek3*.

### Nucleotide diversity at Jek1 and Jek3 within barley

We analyzed genome fragments of *Jek1* and *Jek3* (PCR amplification of approximately 850 bp for both sequences covering parts of the 5′ and 3′ untranslated regions, the full open reading frame and the intron) for 485 diverse *Hordeum* accessions comprising wild barley, landraces and modern cultivated varieties (Table S2). *Jek1* was present in 216 accessions and *Jek3* in 270 accessions. Both sequences were amplified from accession FT588, which was probably heterozygous at this locus and therefore excluded from the analysis. *Jek3* was carried by 63.7% of the wild barley accessions, whereas among the modern cultivars, *Jek1* and *Jek3* were equally frequent (140 carried *Jek1* and 139 *Jek3*). There was no association between *Jek* alleles and any major phenotypic character or geographic origin (Table S2). Here, 58.6% of modern cultivars carrying *Jek1* were of the two‐rowed spike types and 40.7% were spring types. Of *Jek3*‐containing cultivars, 46.0% produced two‐rowed spikes and 54.0% were of the spring type (Table S2).

**Figure 4:**
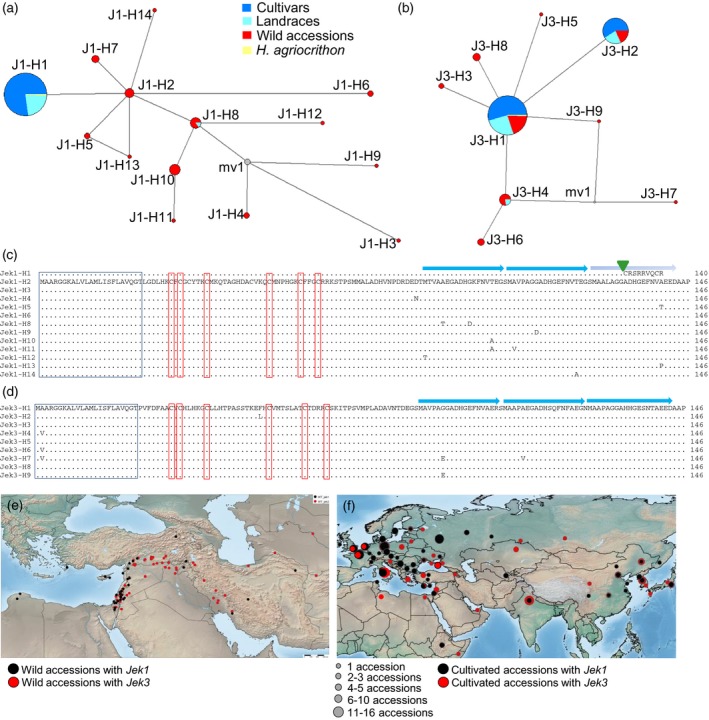
Natural nucleotide diversity at *Jek1* and *Jek3*. (a, b) Median‐joining networks for *Jek1* (a) and *Jek3* (b). (c, d) Haplotype diversity at the peptide level for Jek1 (c), Jek3 (d). The single nucleotide deletion distinguishing J1‐H1 from J1‐H2 haplotypes is shown by a green triangle. The signal peptide (boxed in blue) and the six conserved cysteine residues (boxed in red) are present in all the haplotypes. The direct repeats at the C‐termini of Jek1 and Jek3 are indicated by blue arrows. (e) Geographical provenance of *Jek1* and *Jek3* among wild barley accessions. (f) Geographical provenance of *Jek1* and *Jek3* among domesticated barley accessions.

A more detailed analysis of the *Jek1* sequence revealed 14 haplotypes (Figures 4a and S5a). The most frequent haplotype J1‐H1 was found only in domesticated barleys (140 cultivars, 40 landraces and one feral *Hordeum agriocrithon*). This haplotype was identical to the previously described *Jekyll* sequence (Radchuk *et al*., 2006) and differs from the other *Jek1* haplotypes by a single nucleotide (guanine) insertion in the coding sequence at position 522 downstream of the ATG start codon (Figure S5a). The insertion of this base has generated a frame shift that altered the C‐terminus, reducing the number of direct repeats to two (Figure 4c). While domesticated barleys harbor only two haplotypes for *Jek1* (J1‐H1, J1‐H8), from which J1‐H1 is most frequent and unique for domesticated barleys, altogether 13 *Jek1* haplotypes were found for wild barleys (Figure 4a,c). This situation for *Jek1* indicated a genetic bottleneck during domestication history, in which J1‐H1 was introduced in breeding. This finding is supported by significant negative Tajima's D values at *Jek1* considering all 215 accessions (Table S3). Haplotype J1‐H2 was found to be most closely related to J1‐H1 and can be considered as its ancestral haplotype (Figure S5a). J1‐H2 was found in three wild barleys from the eastern Mediterranean (Cyprus, Israel, Jordan), possibly indicating the region of origin for J1‐H1. In general, most wild barleys carrying *Jek1* haplotypes were found in the western part of the Fertile Crescent, with a few exceptions, coming mainly from Iran (Figure 4e).

In addition to the J1‐H1 haplotype, the other *Jek1* haplotypes encoded a C‐terminus, similar to the *Jek3* C‐terminus (Figure 4c,d), and contained three almost perfect repeats also in the *Jek1* protein. The haplotypes J1‐H6, J1‐H7, J1‐H8 and J1‐H13 differed from J1‐H2 only by synonymous substitutions and the other haplotypes exhibited one or several non‐synonymous substitutions (Figure S5a). However, the signal peptide and the cysteine positions in the middle region of the protein were highly conserved among all haplotypes.

Landraces harboring haplotype J1‐H1 were collected in northern Africa, south‐eastern Europe, eastern Anatolia and Central Asia (Figure S6b). It is interesting to note that all analyzed Ethiopian accessions contained only *Jek1* (Figure S6b,c and Table S2).

The *Jek3* sequence was represented by nine haplotypes (Figures 4b,d and S5b). The haplotypes J3‐H1 (75% of all accessions with *Jek3*) and J3‐H2 (17%) were the most frequent. All haplotypes were represented in wild barley, three in landraces and two (J3‐H1, J3‐H2) among the cultivars, also suggesting a genetic bottleneck at *Jek3* for domesticated barley. Most of the nucleotide variants were synonymous (Figures 4d and S5b). Landraces carrying the J3‐H1 haplotype were found in Eastern Europe and Asia from Turkey to Nepal (Figure S6c). Cultivars with J3‐H1 were widespread in Europe, western Asia and China, Korea but not Japan (Figure 6c). Haplotype J3‐H2 showed a more eastern distribution range and was found in seven wild barleys (one in each Israel and Iraq, two in Turkey and three in Iran), 10 six‐rowed landraces were collected between Iraq and China, as well as in 27 cultivars mainly originating from the Korean peninsula, China and Japan (Figure S6c). Domesticated barleys carrying J3‐H2 produced only six‐rowed or intermedium‐type spikes. It is interesting that wild barleys harboring *Jek3* were widely distributed over the whole Fertile Crescent, while wild barleys with *Jek1* mainly occurred in the Levantine corridor (Figure 4e).

Furthermore, we analyzed the relationship of the *Jek1*/*Jek3* locus to the major genes responsible for traits modified by domestication (rachis fragility, photoperiod responsiveness, and heading time) (Kilian *et al*., 2009) in barley such as *non‐brittle rachis* (*btr*), *Photoperiod‐H1* (*Ppd‐H1*) and *centroradialis* (*HvCEN*). The non‐brittle rachis trait is controlled by two complementary and tightly linked genes *btr1* or *btr2*, located on the same chromosome 3H as the *Jek1*/*Jek3* locus (Pourkheirandish *et al*., 2015). Both non‐brittle *btr1* and *btr2* genotypes carry all domesticated *Jek1* and *Jek3* haplotypes (Table S2). Considering the distribution of *Jek1*/*Jek3*,* HvCEN*,* btr1*/*btr2* and *Ppd‐H1* haplotypes, 15 allele combinations were identified among domesticated barleys (Table S4). Analysis of *Jek1*/*Jek3*,* HvCen* and *Ppd‐H1* alleles in the accessions representing closest wild relatives to *btr1* and *btr2* genotypes (FT262, FT566, FT567, FT514 and FT621) has revealed that J3‐H1, alanine‐containing *HvCen* haplotypes I or III, and photoperiod‐responsive haplotypes were combined (Table S4) and therefore suggested the ancestral state.

To summarize, *Jek1* and *Jek3* are strongly conserved alleles, both being almost equally frequent in domesticated as well as in wild barleys. Domesticated barleys harbored either one *Jek1* haplotype (J1‐H1) or one of three *Jek3* haplotypes (J3‐H1, J3‐H2 or J3‐H4). These findings in the *Hordeum* lineage prompted us to investigate the evolution of *Jek* genes in other species of the grass family.

### Jek genes are restricted to Triticeae and Bromeae species

**Figure 5:**
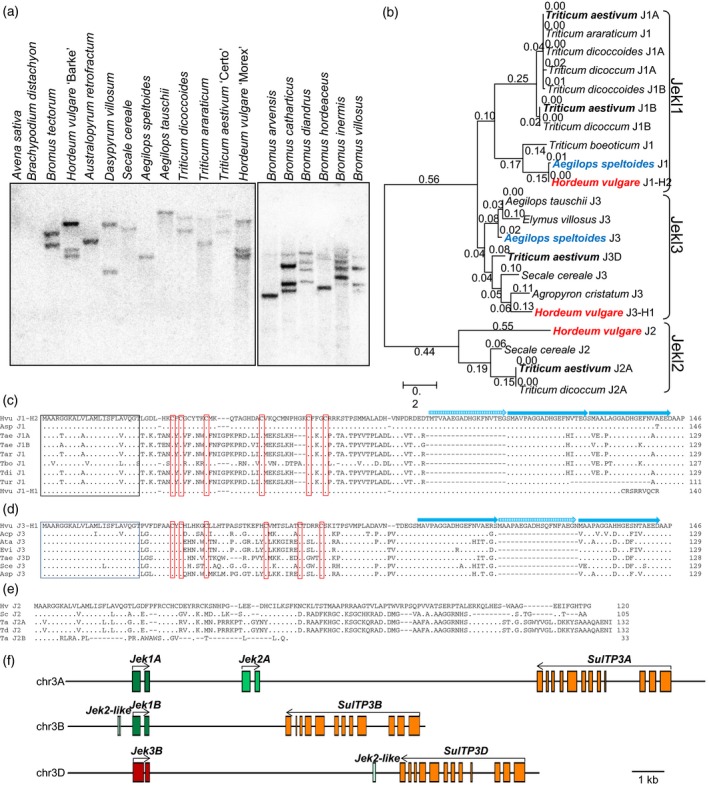
The distribution of *Jek*‐like sequences in species of the grass tribes Triticeae and Bromeae. (a) Southern blot hybridization of genomic DNA extracted from species of the tribes Triticeae (*left‐hand panel*) and Bromeae (*right‐hand panel*), using *Jek1*/*Jek3* conserved region as the probe. (b) Bootstrap consensus tree of Jekyll protein sequences from selected Triticeae species. The maximum‐likelihood method was used to construct this tree with 1000 replicate bootstrap support. The variants found in barley cv. Barke and Morex are indicated in red, those present in *Ae. speltoides* in blue and three Jek homeologs present in one bread wheat genotype in bold. (c–e) Comparison of the deduced amino acid sequences of Jek1 (c), Jek3 (d) and Jek2 (e) from selected Triticeae species. The signal peptide (boxed in black) and the six cysteine residues (boxed in red) are present in each of the Jek1/Jek3 sequences. C‐terminal repeats are indicated by blue arrows. Asp, *Aegilops speltoides*; Ata, *Aegilops tauschii*; Acr, *Agropyron sristatum*; Evi, *Elymus villosus*; Hvu, *Hordeum vulgare*; J1, Jek1; J2, Jek2; J3, Jek3; Sce, *Secale cereale*; Tae, *Triticum aestivum*; Tar, *T. araraticum*; Tbo, *T. boeoticum*; Tdi, *T. dicoccum*; Tds, *T. dicoccoides*.

On the basis of Southern blot hybridization profiles generated using a conserved fragment of *Jek1* as a probe, we identified *Jek*‐like sequences in the genomes of species of the closely related Bromeae and Triticeae tribes (Figure 5a,b) but not in more distantly related *Avena sativa* (Poeae tribe), *Brachypodium distachyon* (Brachypodieae), *Oryza sativa* (Oryzeae) or *Zea mays* (Andropogoneae) (Figure S7; Radchuk *et al*., 2006). A BLAST‐based search in the fully sequenced genomes of *Brachypodium*, rice, sorghum, and maize confirmed the absence of *Jek*‐like sequences in these species. Both *Jek1* and *Jek3* sequences could be amplified from a number of Triticeae species (Figure 5b–d). All of these were deduced to encode proteins that featured the conserved signal peptides and the six conserved cysteine residues, but exhibited great diversity in their central portion (Figure 5c,d). While barley *Jek3* and *Jek1* haplotypes, other than J1‐H1, encoded a protein with three repeats at their C‐terminus, most of the non‐barley *Jek*‐like proteins featured only two C‐terminal repeats. The genome of the tetraploid domesticated emmer wheat (*T. dicoccum*, BBAA genome) as well as its ancestor wild emmer (*T. dicoccoides*) included two *Jek1* copies (*Jek1A* and *Jek1B*, belonging to A and B genome, correspondingly) (Figure 5b,c). The hexaploid bread wheat (*T. aestivum*, BBAADD genome) included three homeologs: two *Jek1* (*Jek1A* and *Jek1B*) genes that were both very similar to the corresponding genes of *T. dicoccum*, and one *Jek3D* (Figure 5b–f). This result is consistent with the domestication history of bread wheat in which domesticated emmer (*T. dicoccum*, BBAA) was hybridized with wild *Ae. tauschii* (DD – the D genome donor of wheat) to produce bread wheat (Peng *et al*., 2011). The *Ae. tauschii* carries a copy of *Jek3* rather than *Jek1* (Figure 5b). Copies of *Jek2* were identified in rye, emmer and bread wheats (Figure 5b,e) further confirming that *Jek2* is another member of the *Jek* family within the Triticeae. However, unlike the *Jek1/Jek3* homeologs, only one fully sized *Jek2* copy was found in both tetraploid and hexaploid wheats.

The genomic region on wheat chromosome 3A housing the *Jek* genes (Figure 5f; International Wheat Genome Sequencing Consortium, 2018) resembled that on barley chromosome 3H (Figure 1d) and harbored *Jek1A*,* Jek2A*, and *SulTP3A*, but the physical distance between *Jek2A* and *SulTP3A* was much longer in wheat than in barley. Wheat chromosome 3B lacked a copy of *Jek2*, so that the distance between *Jek1B* and *SulTP3*B was substantially shortened putatively by deletion. On chromosome 3D, a small fragment of a *Jek2*‐like sequence still remained present, indicative of an independent deletion event (Figure 5f).

To investigate whether both *Jek1* and *Jek3* variants were present in other Triticeae species than barley, we analyzed 10 accessions of wild *Aegilops speltoides* [S genome, closest wild relative to the B genome of bread wheat (Kilian *et al*., 2007)] of different origins. Identical *Jek1* sequences were amplified from four accessions and formed a single haplotype, which encoded a protein that was almost identical (99.3%, one amino acid exchange) to Jek1‐H2 in barley (Figure S8a). The *Jek3* sequence was amplified from six accessions representing five haplotypes (Figure S8b). The number of analyzed samples did not allow deduction of geographical preference in the distribution of *Jek1*/*Jek3* allelic variants for *Ae. speltoides* (Figure S8c).

### Features of grain anatomy peculiar to Triticeae and Bromeae species

**Figure 6:**
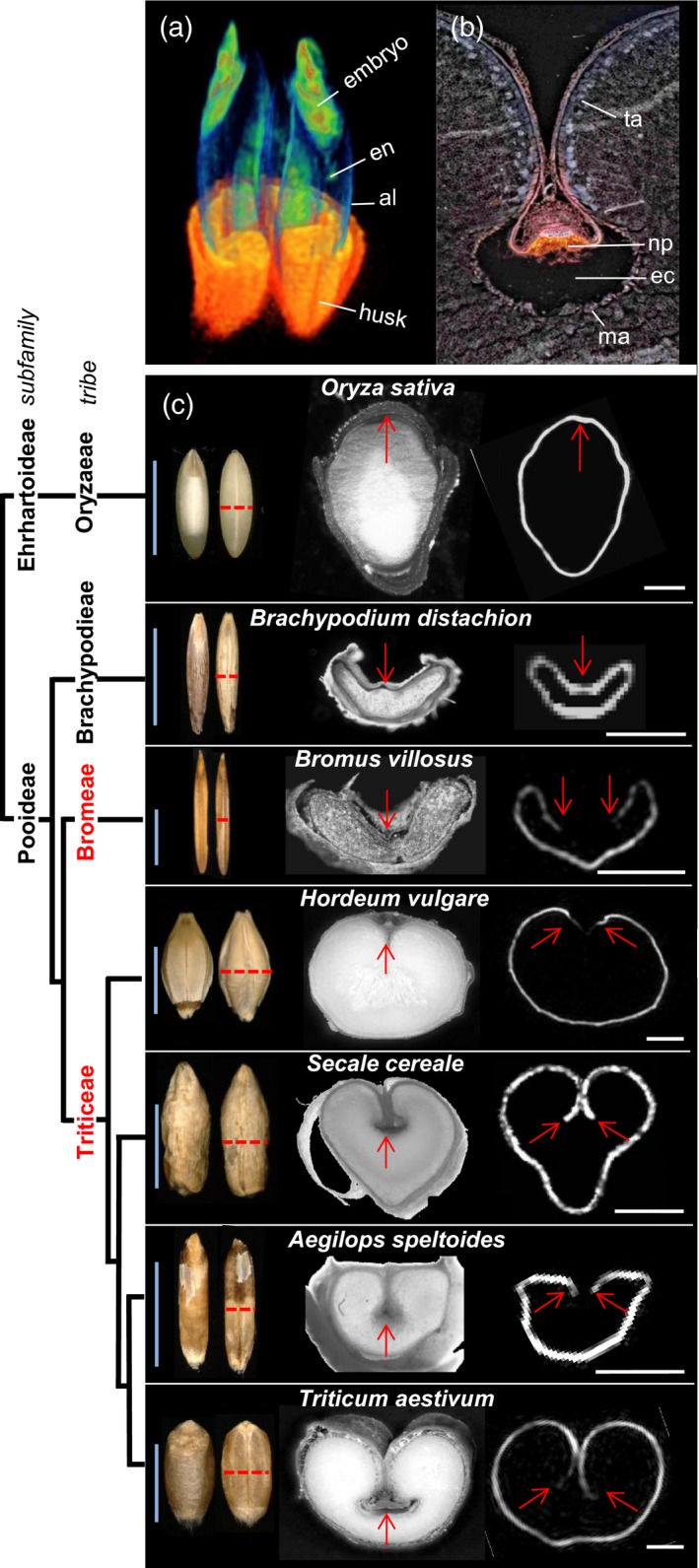
Nuclear magnetic resonance imaging of the grains of the representative species from Triticeae, Bromeae, Brachypodieae and Orizeae tribes. (a) An intact *Aegilops speltoides* spikelet harbors two grains, each covered by the lemma and palea together comprising a husk (orange). The site of lipid deposition within the embryo and aleurone is shown by the yellowish‐green color. (b) The structure of the nucellar projection in a mature *Triticum aestivum* grain, as visualized by light microscopy. (c) Dorsal and lateral views of grains (*left‐hand panel*), virtual cross‐sections of the corresponding grains (*middle panel*) and lipid deposition in the endosperm of *Oryza sativa*,* B. distachyon*,* Bromus villosus*,* Hordeum vulgare*,* Secale sereale*,* Aegilops speltoides* and *T. aestivum* grains, as shown by MRI (white signal, *right‐hand panel*). The positions of virtual cross‐sections of grains are shown by dashed red lines. The crease and the nucellar projection are shown by red arrows. The phylogeny of the species investigated is shown to the left (not to evolutionary scale). A more detailed MRI view of rice and wheat grains is shown on Movie S1. Blue bars: 5 mm; white bars: 1 mm. al, aleurone; ec, endosperm cavity; es, endosperm; ma, modified aleurone; np, nucellar projection; ta, typical aleurone.

In the barley grain, *Jek* activity was confined to the nucellus and the nucellar projection (Figure 2d–f). In these tissues, Jek controls terminal cell differentiation, resulting in cell disintegration (Radchuk *et al*., 2006). The maternal nucellar projection together with endosperm transfer cells provided the main conduit for assimilate transfer towards the endosperm in Triticeae grains (Melkus *et al*., 2011; Hands *et al*., 2012). Non‐invasive nuclear magnetic resonance imaging (MRI) (Borisjuk *et al*., 2013), applied to intact mature grains of several Triticeae species, revealed that endosperm lobes were surrounded by a thin layer of lipids accumulated in the aleurone (Figure 6, Figure S9). Endosperm transfer cells, located opposite the nucellar projection, contain little or no lipids as visualized by virtual cross‐sections through the central regions of the grain of *Ae. speltoides*,* B. villosus*,* H. vulgare*,* S. cereale* and *T. aestivum* caryopses (Figure 6c, Figure S9, Movie S1). In contrast, the endosperm of *Brachypodium* and rice was completely enveloped by lipids forming a typical aleurone layer. A build‐up of lipids in the aleurone had the effect of cutting off the transfer of the assimilate to the endosperm. We speculated that the nucellar activity, controlled by Jek, generated assimilate flow towards the endosperm, preventing final differentiation of endosperm transfer cells into lipid‐accumulating aleurone cells. The formation of a pronounced crease region with the nucellar projection and endosperm transfer cells distinguished the species of the tribes Triticeae and Bromeae from Brachypodieae and other more distant taxonomic groups.

## Discussion

### Jek1 and Jek3 are allelic

Although the deduced Jek1 and Jek3 protein sequences are rather different from one another (sharing just over 50% identity), the two genes behave as alleles: firstly, all the homozygous barley entries of the germplasm set carry either *Jek1* or *Jek3*, and not both or neither; secondly, based on their segregation in mapping populations, *Jek1* and *Jek3* co‐located to the same locus on chromosome 3H; thirdly, they occupied the same genomic position in cv. Morex (*Jek3*) and cv. Golden Promise (*Jek1*); at last, only the *Jek3* genomic region was found in the genome of cv. Morex (Mascher *et al*., 2017) while genomes of cv. Barke and cv. Golden Promise contained only the *Jek1* genomic locus (International Barley Genome Sequencing Consortium, 2012). The two variants shared a similar gene structure: their promoter regions are almost identical, both genomic sequences are interrupted by a single, similarly positioned intron, both translation products start with an identical putative signal peptide, share six conserved cysteine residues within the coding sequence and end in a number of direct repeats. The spatial and temporal pattern of *Jek3* transcription in the developing grain also coincides with that of *Jek1*. As is also the case for the Jek1 protein, recombinant Jek3 inhibits the growth of *E. coli* cells. Finally, *Jek3* is able to functionally complement for the deficiency in *Jek1* expression. The *Jek1/Jek3* divergence points out that some differences in gene content, also in other species, might be attributed to the extreme allelic divergence of the same gene, preserving the functionality and making identification of functionally homologous sequences difficult by only sequence comparisons. Recently developed long‐read sequencing methods will definitely uncover more haplotype‐specific features in genomes (Chaisson *et al*., 2015; Shi *et al*., 2016).

One of each *Jek1* or *Jek3* alleles was present in both domesticated and wild barleys without any preference for alleles or haplotypes in relation to morphological traits or geographical distribution. The *Jek1*/*Jek3* locus lies within the confidence interval surrounding an inferred locus influencing grain yield. This is in an agreement with the experimental suppression of *Jek1*, resulting in a substantial drop in fertility and grain size (Radchuk *et al*., 2006, 2012). However, the possible contribution of *Jek1*/*Jek3* for grain yield was observed only in a few evaluated environments and the gene position was located apart from the QTL peak, indicating that the gene‐trait association was spurious. The equally frequent distribution of *Jek1* and *Jek3* among domesticated barleys indicated no selective sweep. As a result, the biological significance of the *Jek1*/*Jek3* polymorphism remained unclear. While there is some possibility that one of the alleles enabled better adaptation to a particular environment, a more realistic scenario is that the current distribution of *Jek1* and *Jek3* alleles and their haplotypes in domesticated barley reflects the domestication history of the crop.

### The distribution of Jek haplotypes confirms multiple independent barley domestication events

Data on distribution of *Jek1/Jek3* haplotypes among wild and domesticated barleys in conjunction with corresponding data for *HvCen* (Comadran *et al*., 2012), *btr1/btr2* (Pourkheirandish *et al*., 2015) and *Ppd‐H1* (Turner *et al*., 2005) haplotypes provide additional insights into the domestication history of barley. Both *Jek1* and *Jek3* gene variants probably evolved *de novo* in the ancestral lineage of the Triticeae and Bromeae tribes and therefore before the beginning of the *Hordeum* speciation. Because both alleles are present in the wild barley genepool that survived the last glacial maximum (Jakob *et al*., 2014), they had been available at the beginning of the domestication process. Three major *Jek* haplotypes (J1‐H1, J3‐H1 and J3‐H2) were found in domesticated barley. The haplotype J1‐H1 is likely to have originated from J1‐H2 by a natural 1‐bp insertional mutation. Because no wild barley harbored the J1‐H1 mutation, the precise geographic region of its emergence remains speculative. Wild relatives that harbor the progenitor haplotype J1‐H2 have been found in Cyprus, Israel, and Jordan. This finding fits well with the predicted region of origin of the non‐brittle *btr1* phenotype (Pourkheirandish *et al*., 2015). Only the J1‐H1 haplotype was found in Ethiopia, where the *btr1* predominates as well (98%) (Table S2; Pourkheirandish *et al*., 2015) indicating early separation of the gene pool in this region.

Assuming that: (i) *Jek1* and *Jek3* are lineage‐specific for Triticeae and Bromeae; (ii) proline‐containing haplotypes represent the ancestral stage at *HvCen* (Comadran *et al*., 2012); and (iii) proline‐ and alanine‐containing haplotypes at *HvCen* are available in the wild barley genepool (Comadran *et al*., 2012), our findings at *Jek1*/*Jek3*,* HvCen*,* btr1*/*btr2* and *Ppd‐H1* loci clearly speak for multiple independent domestication events in barley that further supporting the most recent studies (Fuller *et al*., 2011, 2012; Comadran *et al*., 2012; Willcox, 2013; Pourkheirandish *et al*., 2015; Civáň and Brown, 2017).

### The significance of Jek1/Jek3 for grass lineage speciation

The emergence of novel genes is a strong driver of speciation. These genes can originate in a number of different ways (Kaessmann, 2010; Chen *et al*., 2013). The *Jekyll* genes appear to have originated *de novo* in the common ancestor of the Triticeae and Bromeae tribes. The birth of novel genes is not necessarily a rare event (Kaessmann, 2010; Ruiz‐Orera *et al*., 2015; Li *et al*., 2016). Similarly, as many new genes that once emerged rapidly become essential for development (Chen *et al*., 2013), Jek plays a pivotal role in sexual reproduction. The presence of Jek1 protein on the surface of pollen grains (Radchuk *et al*., 2012) suggests a function associated with either pollination and/or fertilization. This suggestion is supported by the observation of a major reduction in fertility, when a plant unable to fully express *Jek1* was pollinated to either a *Jek1*‐repressed or a wild type female parent (Radchuk *et al*., 2012). We hypothesize that *Jekyll* emerged during the evolution of the Poaceae as a driver for the separation of the closely related Bromeae and Triticeae lineages from Brachypodieae. Its acquisition was likely to have spurred innovations to the structure/physiology of the grain, specifically by evolving a maternal/filial tissue conduit for transferring assimilate into the endosperm via the nucellar projection and endosperm transfer cells (Melkus *et al*., 2011). Cell death associated with the formation of the endosperm cavity (Radchuk *et al*., 2006) possibly triggers the neighboring aleurone cells to differentiate into endosperm transfer cells. In contrast to the aleurone cells in rice and *B. distachyon* grains, modified aleurone layers that face the nucellar projection in Triticeae and Bromeae grains are unable to accumulate lipids (Figure 6c). Jekyll appears therefore to have become part of the cellular machinery to ensure endosperm development and the accumulation of storage compounds.

Genes involved in sexual reproduction appear to evolve particularly rapidly, with their sequences becoming altered by duplication, deletion, and the divergence of repetitive domains (Fiebig *et al*., 2004; Clark *et al*., 2006). While the tertiary structure of the Jekyll proteins produced by a variety of Triticeae species has remained well conserved, their amino acid composition and the number of C‐terminal repeats differ from species to species. Such an extensive amount of sequence change, which implies a particular rapid evolution of the genes, may well have contributed to speciation within the Triticeae lineage. *Jek2* meanwhile is ubiquitous in barley and is also represented in other Triticeae species; at present, the function of its product is unclear. Even though its transcriptional behavior mirrored that of *Jek1*/*Jek3*,* Jek2* cannot rescue the phenotype induced by knocking down *Jek1* (Radchuk *et al*., 2006).

To conclude, the *Jek1* and *Jek3* genes of the small *Jekyll* family are highly divergent alleles that encode unusual sequentially different but functionally similar proteins. The *Jekyll* family is likely to have emerged *de novo* in the progenitor of the Triticeae and Bromeae tribes and could have contributed to the separation of these tribes from other lineages within the Pooideae.

## Experimental procedures

### Plant materials

For most analyses, 12‐day‐old seedling leaves of domesticated (*Hordeum vulgare* ssp. *vulgare*) and wild barley (*H. vulgare* ssp. *spontaneum*) together with the other species of the Poaceae were used. For seed material or segregation studies, barley plants were grown in a greenhouse under a 16 h light/19°C and 8 h dark/14°C regime during their vegetative phase. The time of fertilization and the developmental stage of caryopses were determined as described (Radchuk *et al*., 2006). For reciprocal cv. Barke × cv. Morex and cv. Morex × N91 crosses, emasculated flowers of isolated maternal spikes were pollinated with pollen of the parental line and vice versa. Hybrid seeds were further grown in F_1_ and F_2_ generations for segregation analysis. For analysis of *Jek1*/*Jek3* nucleotide diversity, 485 wild and domesticated barley accessions were considered and represented 99 wild barleys from 11 countries, two *H. agriocrithon* from two countries, 104 landraces and 279 cultivars from around 50 countries (Table S2).

### DNA and RNA analyses

*Jek1* and *Jek2* sequences were amplified from cDNA prepared from mRNA extracted from the immature grains of cv. Barke, inserted into the pGEM‐T Easy vector (Promega, Mannheim, Germany) and sequenced. Similarly, *Jek3* and *Jek2* sequences were recovered from the grain of cv. Morex. Total genomic DNA was extracted from leaf tissue harvested from 12‐day‐old seedlings following Palotta *et al*. (2000) and used as the template in a PCR with the following primers: 5′‐CTACCAGTAGCACTCTCCCAGTCCC‐3′ (a sequence shared by all three *Jek* sequences) and either 5′‐CAACTTCCGTGGTGTATAACAAAATGAC‐3′ (*Jek1*), 5′‐CAAAGTGGCAGGACATTCACTCATAAC‐3′ (*Jek2*) or 5′‐CATGTGCAGCCCGGACTTTTC‐3′ (*Jek3*). The lengths of the three amplicons were, respectively, 845, 615, and 841 bp. Each 25 μl PCR contained 0.5 μg template, 0.5 U *Taq* DNA polymerase (Roche, Mannheim Germany), 1× buffer, 0.2 mm dNTP and 1 mm of each primer. The amplification profile comprised an initial denaturation step (95°C/4 min), followed by 35 cycles of 95°C/1 min, 58°C/1 min, 72°C/1 min, and was completed by a final extension step (72°C/10 min). The amplicons were electrophoretically separated through a 1% (w/v) agarose gel and visualized by ethidium bromide (EtBr) staining.

To carry out northern blot analyses, total RNA was extracted from developing grains using a Gentra RNA isolation kit (Biozym, Hessisch Oldendorf, Germany) and purified using a DNeasy RNA Isolation kit (Qiagen, Hilden, Germany). A 10 μg aliquot of the resulting RNAs was electrophoretically separated through a 1% (w/v) agarose/formaldehyde gel, blotted and hybridized with labeled copies of either the *Jek1*,* Jek2*, or *Jek3* sequence. Southern blot analyses were performed as described by Radchuk *et al*. (2006).

### Mapping of the Jek genes and QTL analysis

*Jek1* and *Jek3* sequences were mapped based on data obtained from the progenies by PCR with the above‐described primers. Linkage mapping was performed using MAPMAKER v.2.0 (Lander *et al*., 1987) based on Kosambi's (Kosambi, 1944) mapping function and using a minimum LOD score of 3.0 and a maximum recombination frequency of 50%.

The *Jek3* sequences of barley cv. Steptoe and cv. Morex differed by two SNPs that allowed the development of a cleaved amplified polymorphic sequence (CAPS) marker based on a *Sac*I restriction site. For CAPS mapping in the Steptoe/Morex population, PCRs with *Jek3* gene‐specific primers were performed and the PCR products were digested with *Sac*I (Fermentas, Lithuania) for 1 h at 37°C, followed by electrophoretic separation on 1.5% agarose.

To investigate co‐location of *Jek* genes and QTLs for grain yield on a map of Steptoe × Morex and cv. Harrington × cv. Morex mapping populations (https://wheat.pw.usda.gov/ggpages/HxM/), single marker analysis (SMA) and composite interval mapping (CIM) were employed using Windows QTL Cartographer 2.5 (http://statgen.ncsu.edu/qtlcart/WQTLCart.htm). An LOD score ≥3 was used to define a QTL. The proportion of observed phenotypic variation explained due to a QTL was estimated by the coefficient of determination (*R* ^2^). CIM analysis was performed with a 2 cm walk speed and a type 1 error rate of 5%. Intervals of five background markers with a window width of 10 cm were analyzed to control the QTL background effects. To test the relevance of the *Jek1*/*Jek3* locus for grain traits, candidate‐gene association analysis was performed as described (Pasam *et al*., 2012).

### Quantitative RT‐PCR

Quantitative RT‐PCRs (qRT‐PCR) were performed and relative transcript abundances were estimated as described (Tran *et al*., 2014). Primers are listed in Table S5. *Hordeum vulgare* actin gene (HORVU1Hr1G002840.6) was used as a reference. Experiments were run with three or four biological replications and three technical repetitions each.

### *In situ* hybridization

Grains of cv. Morex sampled at various developmental stages were fixed in 50% (v/v) ethanol, 5% (v/v) acetic acid and 3.7% (w/v) formaldehyde overnight at 4°C, dehydrated and embedded in paraffin. Cross‐sections (12 μm) were mounted on a silane‐coated slide (Sigma‐Aldrich, Darmstadt, Germany), and the preparations were de‐waxed, rehydrated and exposed to 2 μg ml^−1^ proteinase K for 30 min at 37°C. Finally, the tissue sections were dehydrated in preparation for the hybridization/immunological detection procedures performed as described (Radchuk *et al*., 2006). The hybridization probe was 1 ng μl^−1^ digoxigenin‐labeled either sense or antisense *Jek3* RNA synthesized from cDNA using either T3 or T7 RNA polymerase (Roche, Mannheim, Germany). The sections were challenged with alkaline phosphatase‐conjugated anti‐digoxigenin antibody and the signal generated was visualized by providing 4‐nitroblue tetrazolium chloride and 5‐bromo‐4‐chloro‐3‐indolyl phosphate (Roche).

### Heterologous expression of *Jek* in *E. coli*

Full‐length sequences encoding both Jek2 and Jek3 were amplified by PCR using primers listed in Table S5 and inserted in frames between the *Bam*HI and *Xho*I cloning sites of the pET23a plasmid (Merck, Darmstadt, Germany). The pJ1 construct containing the open reading frame of *Jek1* was taken from Radchuk *et al*. (2006). Each construct was transformed into *E. coli* strain BL21, and the cells were grown at 37°C for 10 h in Luria‐Bertani medium containing 100 μg ml^−1^ ampicillin and 100 μg ml^−1^ IPTG. Bacterial growth was monitored on an hourly basis by measuring the optical density at 600 nm.

### SNP detection, haplotype analysis and population genetic analysis

DNA sequences were processed with AB DNA Sequencing Analysis Software 5.2 and later manually edited by BioEdit version 7.0.9.0 (Hall, 1999). Sequence alignments were generated with ClustalW, and the allelic haplotypes were defined by DNASP 5.10.01 (Librado and Rozas, 2009). All singletons were confirmed afterwards by an additional three independent amplifications and sequencing. Forty‐four polymorphic positions were detected at *Jek1* that defined 14 haplotypes. At *Jek3*, only 12 polymorphic positions were found that defined nine haplotypes (Figure S5).

Median‐joining networks (Bandelt *et al*., 1999) were constructed for *Jek1* and *Jek3* haplotypes using the programs DNA Alignment 1.3.3.2 and Network 5.0.0.1 (Fluxus Technology Ltd., Clare, Suffolk, UK). Polymorphic sites files were generated in DNASP 5.10.01 (Librado and Rozas, 2009), where gaps were considered.

Population genetic parameters, nucleotide diversity π (Nei, 1987), θ (Watterson, 1975) and Tajima's D (Tajima, 1989), population divergence, were calculated using DNASP 5.10.01 considering the sequenced regions of *Jek1/Jek3* and by grouping the accessions into domesticated, wild and landrace populations (Librado and Rozas, 2009). Loss of nucleotide diversity (*L*_π_) was calculated as *L*_π_, which was calculated using *L*_π_ = 1 − (π_domest_/π_wild_), in which wild and domesticated barley nucleotide diversities were compared (Tenaillon *et al*., 2004).

### Phylogenetic analysis

Protein sequences were aligned using the ClustalX (Jeanmougin *et al*., 1998). The phylogenetic trees were constructed using the maximum‐likelihood methods in MEGA6 with the following option settings: Poisson substitution model, uniform rates, partial deletion for gaps/missing data, 95% site coverage cutoff, strong branch swamp filter and 1000 bootstrap replications.

### MRI‐based visualization of grain structure

MR imaging was performed on a 500 MHz Avance nuclear magnetic resonance (NMR) spectrometer and a 400 MHz Avance 3 HD NMR spectrometer (Bruker Biospin, Rheinstetten, Germany). NMR resonators with an inner diameter of 5 mm were employed as the radio frequency (RF) coil. Acquiring high resolution, frequency‐selective *in vivo* lipid images required an experimental time of around 3.5–16 h, depending on the field of view. Matrix size and field of view were adjusted to achieve a spatial resolution between 50 and 104 μm. In the spin echo sequence, the repetition times (TR) selected were between 500 and 1000 ms, the echo times (TE) were set to the minimal value, namely between 4.4 to 7.9 ms. For optimization of Signal‐to‐Noise Ratio, the datasets were averaged two to eight times. Image processing and analysis were performed using MATLAB software (The MathWorks, Natick, MA, USA).

## Accession numbers

Sequence data from this article can be found in the EMBL/GenBank data libraries under accession numbers: *Aegilops speltoides Jek1*, MK432918; *A. speltoides Jek3*, MK432919; *A. tauschii Jek3*, AET3Gv20681700.1; *Agropyron cristatum Jek3*, MK432920; *Elymus villosus Jek3*, MK432921; *Hordeum vulgare Jek1*, AM261729; *H. vulgare Jek2*, HORVU3Hr1G068150.1; *H. vulgare Jek3*, HORVU3Hr1G068160.1; *HvSulTP3*, HORVU3Hr1G068140.3; *Secale cereale*, MK432922; Triticum *aestivum Jek1A*, TraesCS3A02G288600.1; *T. aestivum Jek1B*, TraesCS3B02G323400.1; *T. aestivum Jek3D*,* TraesCS3D02G288500.1*;* T. aestivum Jek2A*, TraesCS3A02G288700.1; *T. araraticum Jek1* MK432923; *T. boeticum Jek1* MK432924; *T. dicoccoides Jek1A*, TRIDC3AG042600.1; *T. dicoccoides Jek1B*;* T. diccocum Jek1A* MK432925; *T. diccocum Jek1B* MK432926; *T. diccocum Jek2A*, MK432927.

## Conflict of interest

The authors declare no conflicts of interest.

## Supporting information


**Figure S1.** Genomic structure of *Jek* genes.

**Figure S2**. Inheritance and location of *Jek1 and Jek3* sequences.

**Figure S3.** Co‐location of *Jek1 and Jek3* positions, and QTLs for grain yield on 3H chromosome as detected in two barley mapping populations.

**Figure S4**. Expression profiles of *Jek1* and *Jek2* genes in the different tissues micro‐dissected from the developing barley grains of cv. Barke.

**Figure S5.** Nucleotide alignments of haplotype sequences of *Jek1* and *Jek3*.

**Figure S6.** Geographical distribution of wild and cultivated barleys with *Jek1* and *Jek3*.

**Figure S7.** Phylogenetic tree of selected species from the Poaceae family used in the present study and in Radchuk *et al*. (2006).

**Figure S8.** Distribution of *Jek* sequences in *Aegilops speltoides* population.

**Figure S9.** NMR imaging of lipid deposition in grains of selected wild *Triticum* species (a), two cultivars of domesticated *T. aestivum* (b) and five cultivars of *Hordeum vulgare* (c).

**Table S1**. LOD scores and the proportion of observed phenotypic variation (R^2^) for the grain yield QTL assigned to the *Jek* locus on chromosome 3H in the Harrington × Morex and Steptoe × Morex mapping populations.

**Table S2.** List of barley accessions used in nucleotide diversity study.

**Table S3**. Population statistics of re‐sequenced *Jek1*/*Jek3* accessions.

**Table S4.** Jek1/Jek3, HvCEN, btr1/btr2 and Ppd‐H1 allelic combinations in domesticated barleys.

**Table S5**. List of primers used.

**Movie S1**. Comparative *in vivo* visualization of lipid distribution in barley (left) versus rice grains (right) by MRI. The lipid layer is absent in the region corresponding to the nucellar projection in the mature barley grain but present in the rice grain.
