# Supplementary material for: The highly divergent Jekyll genes, required for sexual reproduction, are lineage specific for the related grass tribes Triticeae and Bromeae
Source: Plant J. 2019 May 25;98(6):961–74. doi: 10.1111/tpj.14363 (PMC6851964; doi:10.1111/tpj.14363)
Supplement: Supplementary file 1 — Figure S1. Genomic structure of Jek genes. [file TPJ-98-961-s001.pdf]

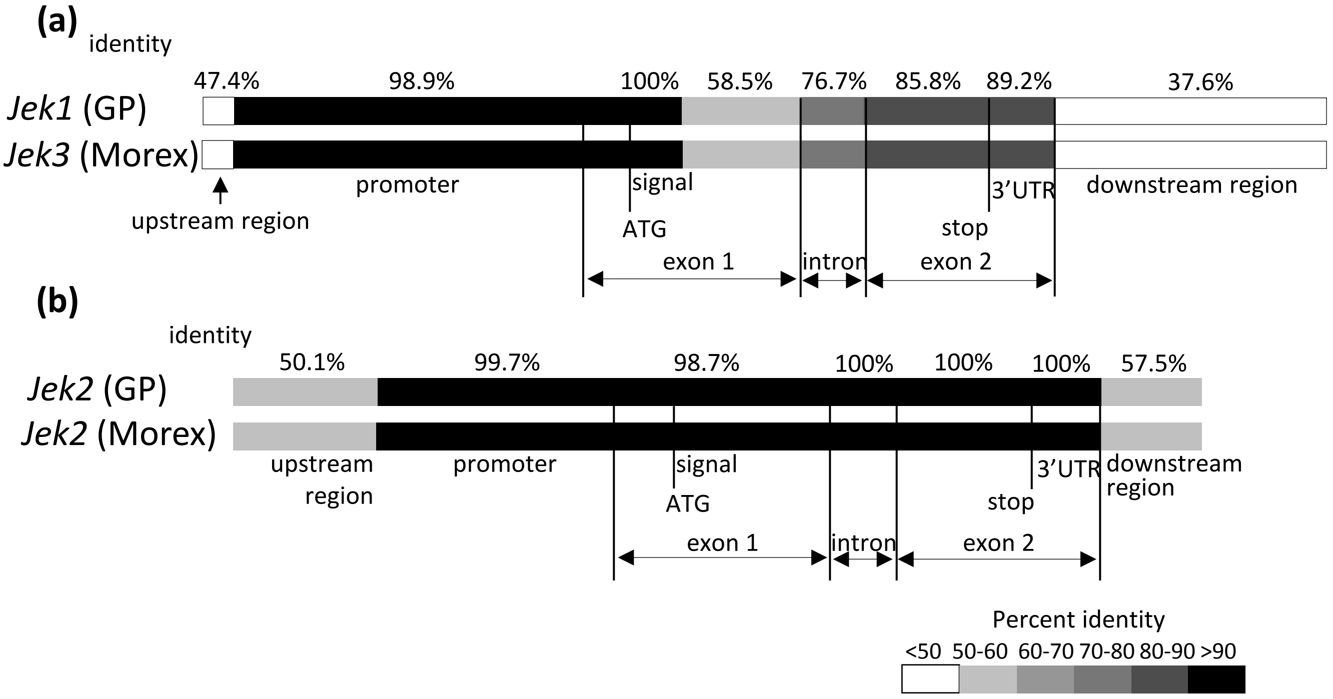

Figure S1. Genomic structure of *Jek* genes. (a) Comparison of genomic fragments containing *Jek1* from Golden Gromise (GP) (contig_81467) and *Jek3* from Morex genotype (contig_42242). (b) Comparison of *Jek2* genomic fragments derived from GP and Morex genotypes. Grey scale (below) represents the percentage of identity. 3'UTR, 3' untranslated region; ATG, translation start; signal, signal peptide; stop, translation stop.
