# Supplementary material for: The highly divergent Jekyll genes, required for sexual reproduction, are lineage specific for the related grass tribes Triticeae and Bromeae
Source: Plant J. 2019 May 25;98(6):961–74. doi: 10.1111/tpj.14363 (PMC6851964; doi:10.1111/tpj.14363)
Supplement: Supplementary file 2 — Figure S2. Inheritance and location of Jek1 and Jek3 sequences. [file TPJ-98-961-s002.pdf]

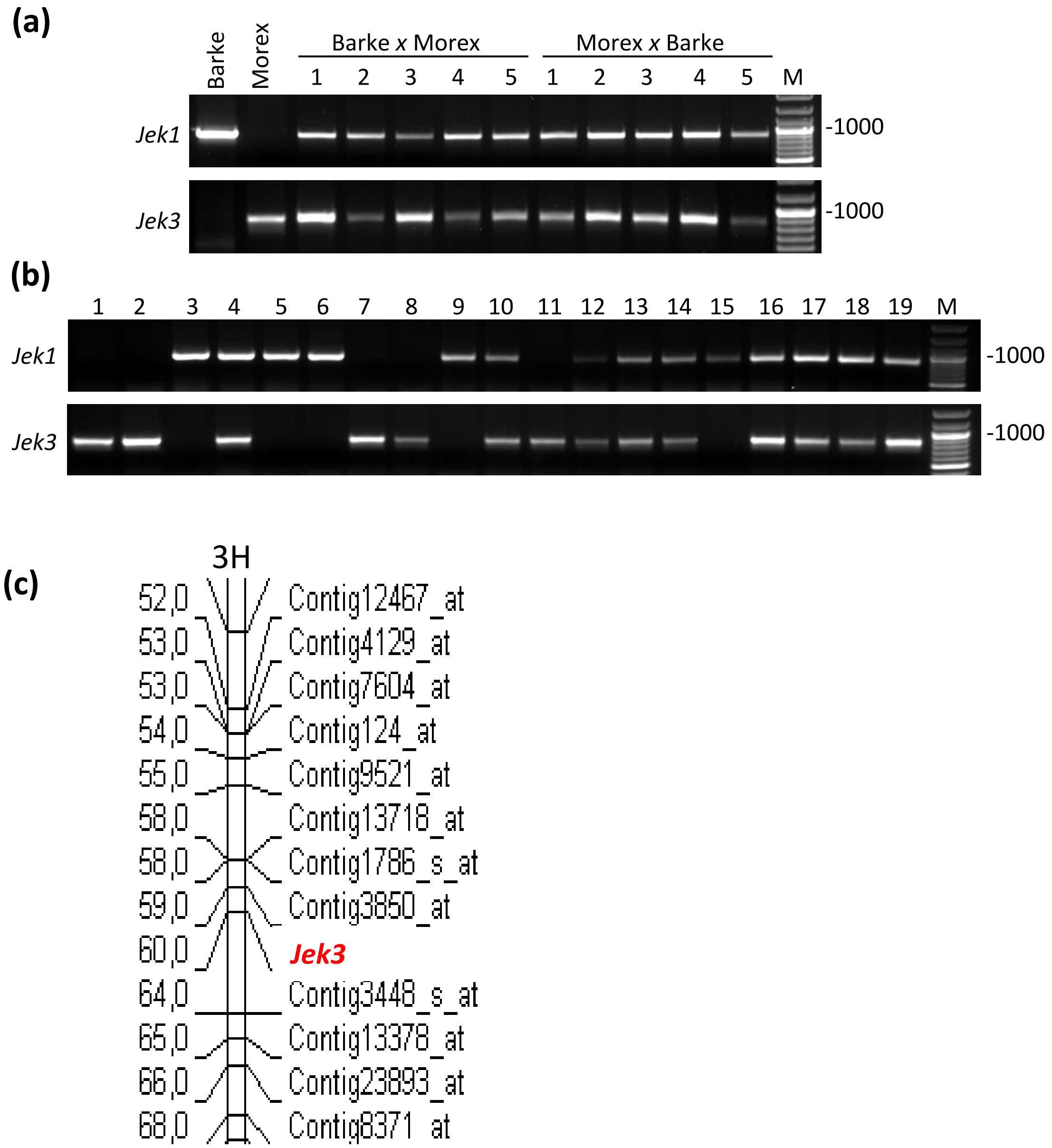

Figure S2. Inheritance and location of *Jek1 and Jek3* sequences. (a) Inheritance of *Jek1* and *Jek3* sequences in F_1 hybrids of Barke *x* Morex cross. (b) Segregation of *Jek1* and *Jek3* sequences in F_2 plants of Barke *x* Morex cross. (c) Location of *Jek3* gene at 3H chromosome on a Steptoe x Morex (both contain *Jek3*) genetic map.
