# Supplementary material for: The highly divergent Jekyll genes, required for sexual reproduction, are lineage specific for the related grass tribes Triticeae and Bromeae
Source: Plant J. 2019 May 25;98(6):961–74. doi: 10.1111/tpj.14363 (PMC6851964; doi:10.1111/tpj.14363)
Supplement: Supplementary file 3 — Figure S3. Co‐location of Jek1 and Jek3 positions, and QTLs for grain yield on 3H chromosome as detected in two barley mapping populations. [file TPJ-98-961-s003.pdf]

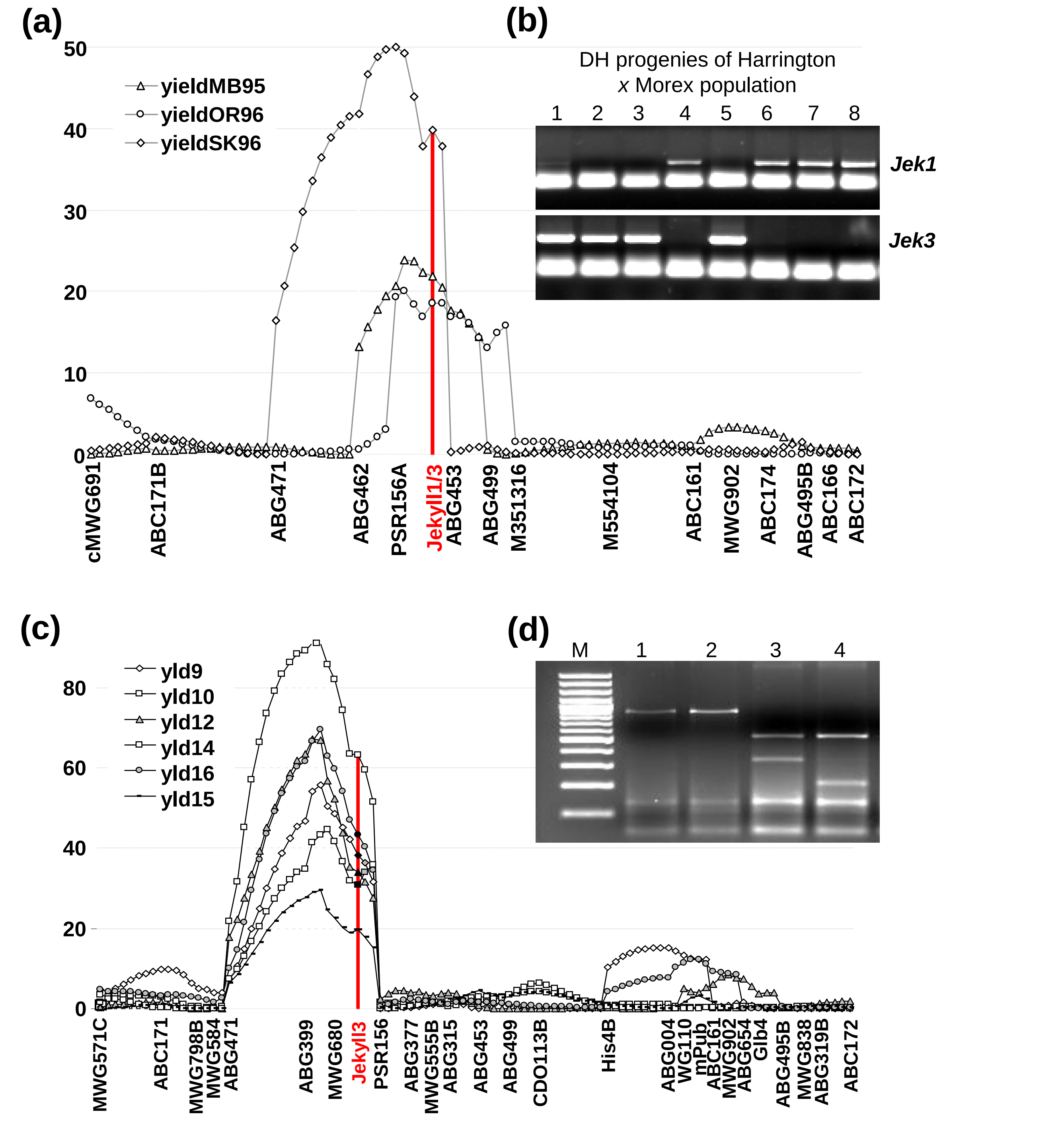


**Figure S3.** Co-location of *Jek1 and Jek3* positions, and QTLs for grain yield on 3H chromosome as detected in two barley mapping populations. (a) Likelihood ratios (LR) for the grain yield QTLs revealed for Harrington *x* Morex cross in three environments. (b) Segregating *Jek1* and *Jek3* sequences among progenies of Harrington x Morex population as revealed by PCR amplification with gene-specific primers. (c) LR for the grain yield QTLs revealed for Steptoe *x* Morex cross in six environments. (d) CAPS marker developed to localize the *Jek1* sequence on the Steptoe x Morex genetic map. M, molecular marker; 1 and 2, amplified Jek3 fragments from Morex (1) and Steptoe (2) correspondingly; 3 and 4, amplified Jek3 fragments digested with *Sac* I enzyme from Morex (3) and Steptoe (4) correspondingly.
