# Supplementary material for: The highly divergent Jekyll genes, required for sexual reproduction, are lineage specific for the related grass tribes Triticeae and Bromeae
Source: Plant J. 2019 May 25;98(6):961–74. doi: 10.1111/tpj.14363 (PMC6851964; doi:10.1111/tpj.14363)
Supplement: Supplementary file 4 — Figure S4. Expression profiles of Jek1 and Jek2 genes in the different tissues micro‐dissected from the developing barley grains of cv. Barke. [file TPJ-98-961-s004.pdf]

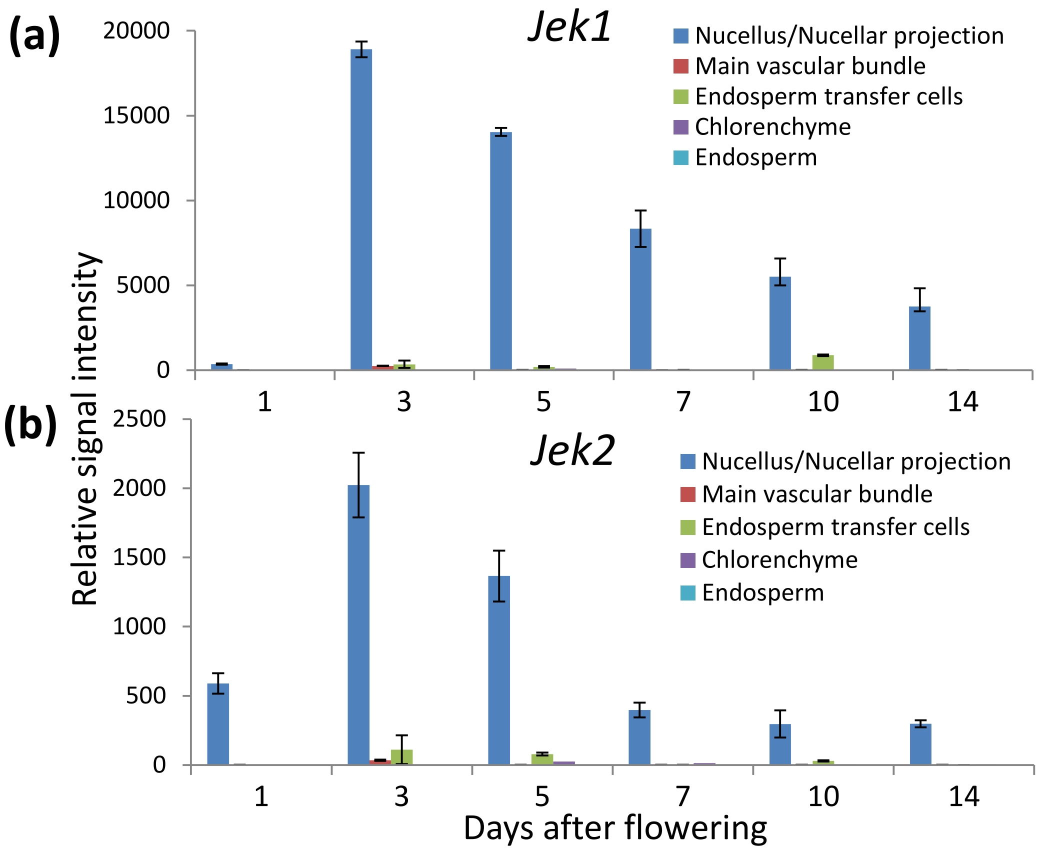

Figure S4. Expression profiles of *Jek1* (a) and *Jek2* (b) genes in the different tissues micro-dissected from the developing barley grains of cv. Barke.
