# Supplementary material for: The highly divergent Jekyll genes, required for sexual reproduction, are lineage specific for the related grass tribes Triticeae and Bromeae
Source: Plant J. 2019 May 25;98(6):961–74. doi: 10.1111/tpj.14363 (PMC6851964; doi:10.1111/tpj.14363)
Supplement: Supplementary file 5 — Figure S5. Nucleotide alignments of haplotype sequences of Jek1 and Jek3. [file TPJ-98-961-s005.pdf]

A

```
                                      5' URF
Jek1-H1   CCAGTAGCACTCTCCCAGTCCCAGTGTGACTTGCAAGCTCGATCGCGCAATGGCGGCTCGCGGTGGGAAGGCCCTGGTCCTGGCAATGCTGATCTCCTTCCTCGCGGTCCAGGGGACCCTTGGGGATCTCCACAAGTGCT 140
Jek1-H2   ............................................................................................................................................ 140
Jek1-H3   ....................................................................................C....................................................... 140
Jek1-H4   ....................................................................................C....................................................... 140
Jek1-H5   ............................................................................................................................................ 140
Jek1-H6   ............................................................................................................................................ 140
Jek1-H7   ............................................................................................................................................ 140
Jek1-H8   ...........................................T........................................C....................................................... 140
Jek1-H9   ....................................................................................C....................................................... 140
Jek1-H10  ....................................................................................C....................................................... 140
Jek1-H11  ....................................................................................C....................................................... 140
Jek1-H12  ....................................................................................C....................................................... 140
Jek1-H13  ............................................................................................................................................ 140
Jek1-H14  ............................................................................................................................................ 140

Jek1-H1   TCTGCGGGTGCTACACCAAGTGCATGAAGCAAACGGCGGGCCACGATGCCTGCGTGAAGCAGTGCATGAACCCTCATGGCAAGTGTTTCTTTGGGTGCCGGAGGAAGAGTACTCCCTCCATGATGGCATTGGCCGATCAT 280
Jek1-H2   ............................................................................................................................................ 280
Jek1-H3   ............................................................................................................................................ 280
Jek1-H4   ............................................................................................................................................ 280
Jek1-H5   ............................................................................................................................................ 280
Jek1-H6   ............................................................................................................................................ 280
Jek1-H7   ............................................................................................................................................ 280
Jek1-H8   ............................................................................................................................................ 280
Jek1-H9   ............................................................................................................................................ 280
Jek1-H10  ............................................................................................................................................ 280
Jek1-H11  ............................................................................................................................................ 280
Jek1-H12  ............................................................................................................................................ 280
Jek1-H13  ............................................................................................................................................ 280
Jek1-H14  ............................................................................................................................................ 280

Jek1-H1   GTCAACCCAGGTGAGTTATATCATTCCCGCATGCAAAGTGTCTGCGAATGTATGTATAGTATAGTTCGTGTGTTTTCCGCAAGTTTGTTTGTCT--------------------------GACCTGTCGCTGCCTTTCCC 394
Jek1-H2   ..............................................................................................--------------------------.................... 394
Jek1-H3   ..............................................................................................--------------------------.................... 394
Jek1-H4   ........T.....................................................................................--------------------------.................... 394
Jek1-H5   ..............................................................................................--------------------------.................... 394
Jek1-H6   ..............................................................................................TTTTTTTTAGACAAGTTTGTTTGTCT.................... 420
Jek1-H7   ..............................................................................................--------------------------...............A.... 394
Jek1-H8   ........T.....................................................................................--------------------------.................... 394
Jek1-H9   ........T.....................................................................................--------------------------.................... 394
Jek1-H10  ..............................................................................................--------------------------.................... 394
Jek1-H11  ..............................................................................................--------------------------.................... 394
Jek1-H12  ..............................................................................................--------------------------.................... 394
Jek1-H13  ..............................................................................................--------------------------.................... 394
Jek1-H14  ..............................................................................................--------------------------.................... 394
```

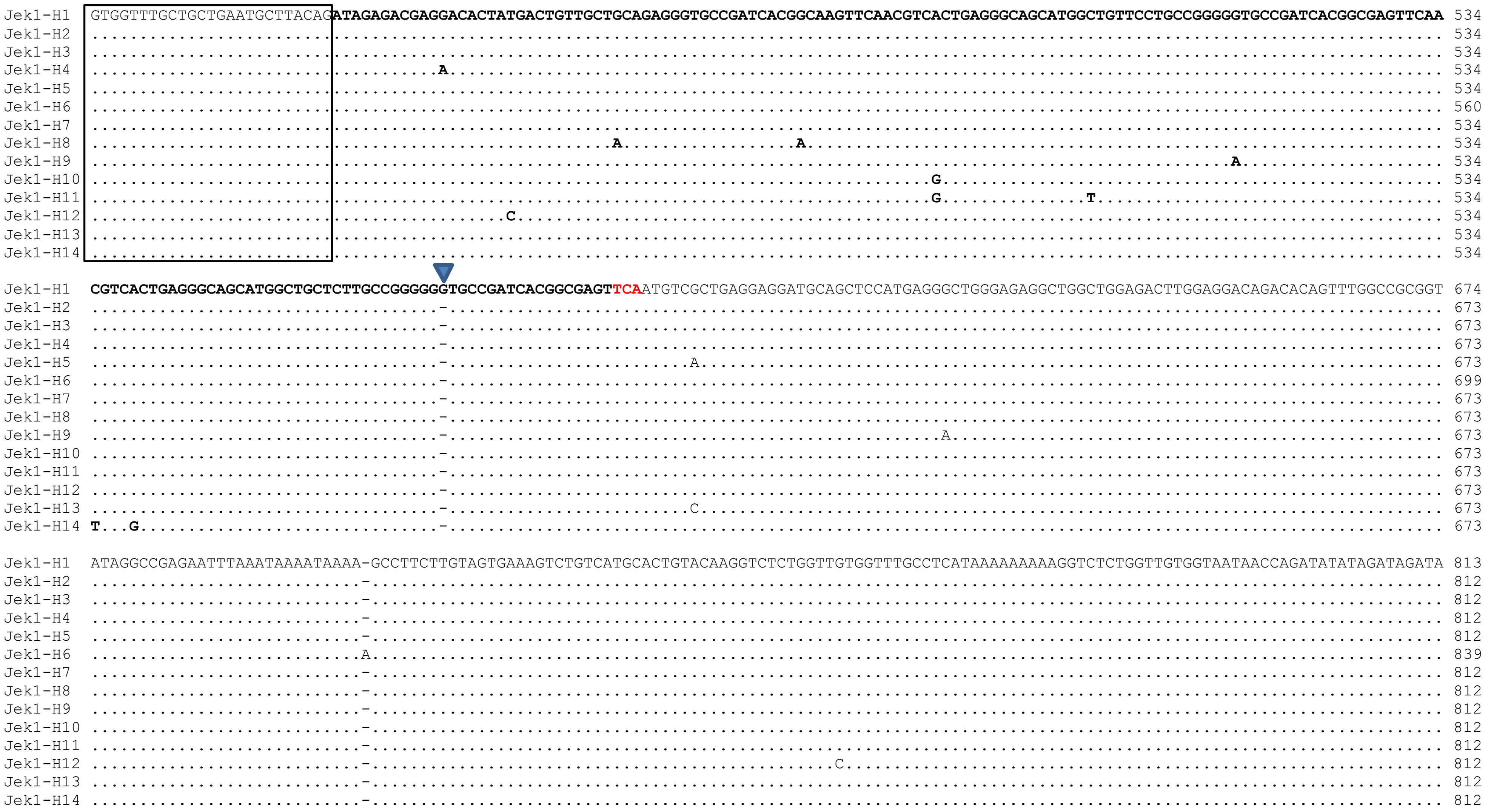

Jek1-H1 GTGGTTTGCTGCTGAATGCTTACAGATAGAGACGAGGACACTATGACTGTTGCTGCAGAGGGTGCCGATCACGGCAAGTTCAACGTCACTGAGGGCAGCATGGCTGTTCCTGCCGGGGGTGCCGATCACGGCGAGTTCAA 534
Jek1-H1 CGTCACTGAGGGCAGCATGGCTGCTCTTGCCGGGGGGTGCCGATCACGGCGAGTTCAATGTCGCTGAGGAGGATGCAGCTCCATGAGGGCTGGGAGAGGCTGGCTGGAGACTTGGAGGACAGACACAGTTTGGCCGCGGT 674
Jek1-H1 ATAGGCCGAGAATTTAAATAAAATAAAA-GCCTTCTTGTAGTGAAAGTCTGTCATGCACTGTACAAGGTCTCTGGTTGTGGTTTGCCTCATAAAAAAAAAGGTCTCTGGTTGTGGTAATAACCAGATATATAGATAGATA 813

```
Jek1-H1   ACTGCAGTGCGTACCATGTGCTTGTCATTTTGT 846
Jek1-H2   ................................. 845
Jek1-H3   ................................. 845
Jek1-H4   ................................. 845
Jek1-H5   ................................. 845
Jek1-H6   ................................. 872
Jek1-H7   ................................. 845
Jek1-H8   ................................. 845
Jek1-H9   ................................. 845
Jek1-H10  ................................. 845
Jek1-H11  ................................. 845
Jek1-H12  ................................. 845
Jek1-H13  ................................. 845
Jek1-H14  ................................. 845
```

B

```
Jek3-H1   TCCACATCCCCACCTCGGCCTCCTACCACTACCAGTAGCACTCTCCCAGTCCCAGTGTGACTTGCAAGCTCGATCGCGCAATGGCGGCTCGCGGTGGGAAGGCCCTGGTCCTGGCCATGCTGATCTCCTTCCTCGCGGTC 140
Jek3-H2   ............................................................................................................................................ 140
Jek3-H3   ............................................................................................................................................ 140
Jek3-H4   ....................................................................................T....................................................... 140
Jek3-H5   ............................................................................................................................................ 140
Jek3-H6   ....................................................................................T....................................................... 140
Jek3-H7   ....................................................................................T....................................................... 140
Jek3-H8   ............................................................................................................................................ 140
Jek3-H9   ............................................................................................................................................ 140

Jek3-H1   CAAGGAACGCCGGTGTTTGATTTCGCCGCGTGCTACTGCCACCTCCACAAGGGGTGCCTATTACACACGCCAGCTAGCAGTACCAAGGAGTTCCACTGCGTGATGACAAGCCTCGCAACATGTACTGATCGGAGGTGCTC 280
Jek3-H2   ..G.......................................................................................C................................................. 280
Jek3-H3   ........................................................T................................................................................... 280
Jek3-H4   ............................................................................................................................................ 280
Jek3-H5   ............................................................................................................................................ 280
Jek3-H6   ............................................................................................................................................ 280
Jek3-H7   ............................................................................................................................................ 280
Jek3-H8   ............................................................................................................................................ 280
Jek3-H9   ............................................................................................................................................ 280

Jek3-H1   GAAGATCACTCCTTCCGTGATGCCACTGGCCGATGCTGTCAACACCGGTGAGTTATATCGTTCCCGCGTTAGAGCTCAGTGTCAGGTGCCTGAGCATGTACTGCATATTATACTAGTATATGTATGTGTGTTTTCCGAAA 420
Jek3-H2   ............................................................................................................................................ 420
Jek3-H3   ............................................................................................................................................ 420
Jek3-H4   ............................................................................................................................................ 420
Jek3-H5   ............................................................................................................................................ 420
Jek3-H6   ............................................................................................................................................ 420
Jek3-H7   ............................................................................................................................................ 420
Jek3-H8   ............................................................................................................................................ 420
Jek3-H9   ............................................................................................................................................ 420
```

```
Jek3-H1  GTTTGTTTGTCTGACCTGTGGTTTGCTGCTGAATGCTTACACTTACAGACGAGGGTAGCATGGCCGTTCCTGCCGGGGGTGCCGATCACGGCGAGTTCAACGTCGCTGAGCGCAGCATGGCTGCTCCTGCCGAGGGTGCC 560
Jek3-H2  ............................................................................................................................................ 560
Jek3-H3  ............................................................................................................................................ 560
Jek3-H4  ............................................................................................................................................ 560
Jek3-H5  ............................................................................................................................................ 560
Jek3-H6  ............................................................................................................................................ 560
Jek3-H7  ...........................................................................A.....................................................T.......... 560
Jek3-H8  ............................................................................................................................................ 560
Jek3-H9  ...........................................................................A................................................................ 560

Jek3-H1  GATCACAGCCAGTTCAACTTCGCTGAGGGCAACATGGCTGCTCCTGCCGGGGGTGCCCATCACGGCGAGTCCAACACCGCTGAAGAGGATGCAGCTCCATGAGGGCCGCGAGAGGCTGGCTGGAGACTTGGACGACAGAC 700
Jek3-H2  ............................................................................................................................................ 700
Jek3-H3  ............................................................................................................................................ 700
Jek3-H4  ............................................................................................................................................ 700
Jek3-H5  ............................................................................................................................................ 700
Jek3-H6  .................T.......................................................................................................................... 700
Jek3-H7  ............................................................................................................................................ 700
Jek3-H8  ............................................................................................................................................ 700
Jek3-H9  ............................................................................................................................................ 700

Jek3-H1  ACAGTTTGGCCATGCTATAGGCCGAGAAACTCTTTAAAATAAAAGCGTTCTTGTAGTAAAAGTCTGTCATGCACTGTACAAGATCTCTGGCTGTGGTACTAATAACTATAGATATACATAAATACCAGGGCCGGGCCTAC 840
Jek3-H2  ............................................................................................................................................ 840
Jek3-H3  ............................................................................................................................................ 840
Jek3-H4  ............................................................................................................................................ 840
Jek3-H5  .......................................................C..............................G..................................................... 840
Jek3-H6  ............................................................................................................................................ 840
Jek3-H7  .............................................................................................................................G.............. 840
Jek3-H8  .................................................................................A.......................................................... 840
Jek3-H9  ............................................................................................................................................ 840

Jek3-H1  AGTGGTGTGAA 851
Jek3-H2  ........... 851
Jek3-H3  ........... 851
Jek3-H4  ........... 851
Jek3-H5  ........... 851
Jek3-H6  ........... 851
Jek3-H7  ........... 851
Jek3-H8  ........... 851
Jek3-H9  ........... 851
```

Fig. S5 Nucleotide alignments of haplotype sequences of *Jek1* (A) and *Jek3* (B). Protein-encoding sequences are shown in bolt, ATG start and stop codons are written in red. Sequences encoding putative peptide signals are labeled by red boxes, introns are labelled by black boxes.
