# Supplementary material for: The highly divergent Jekyll genes, required for sexual reproduction, are lineage specific for the related grass tribes Triticeae and Bromeae
Source: Plant J. 2019 May 25;98(6):961–74. doi: 10.1111/tpj.14363 (PMC6851964; doi:10.1111/tpj.14363)
Supplement: Supplementary file 6 — Figure S6. Geographical distribution of wild and cultivated barleys with Jek1 and Jek3. [file TPJ-98-961-s006.pdf]

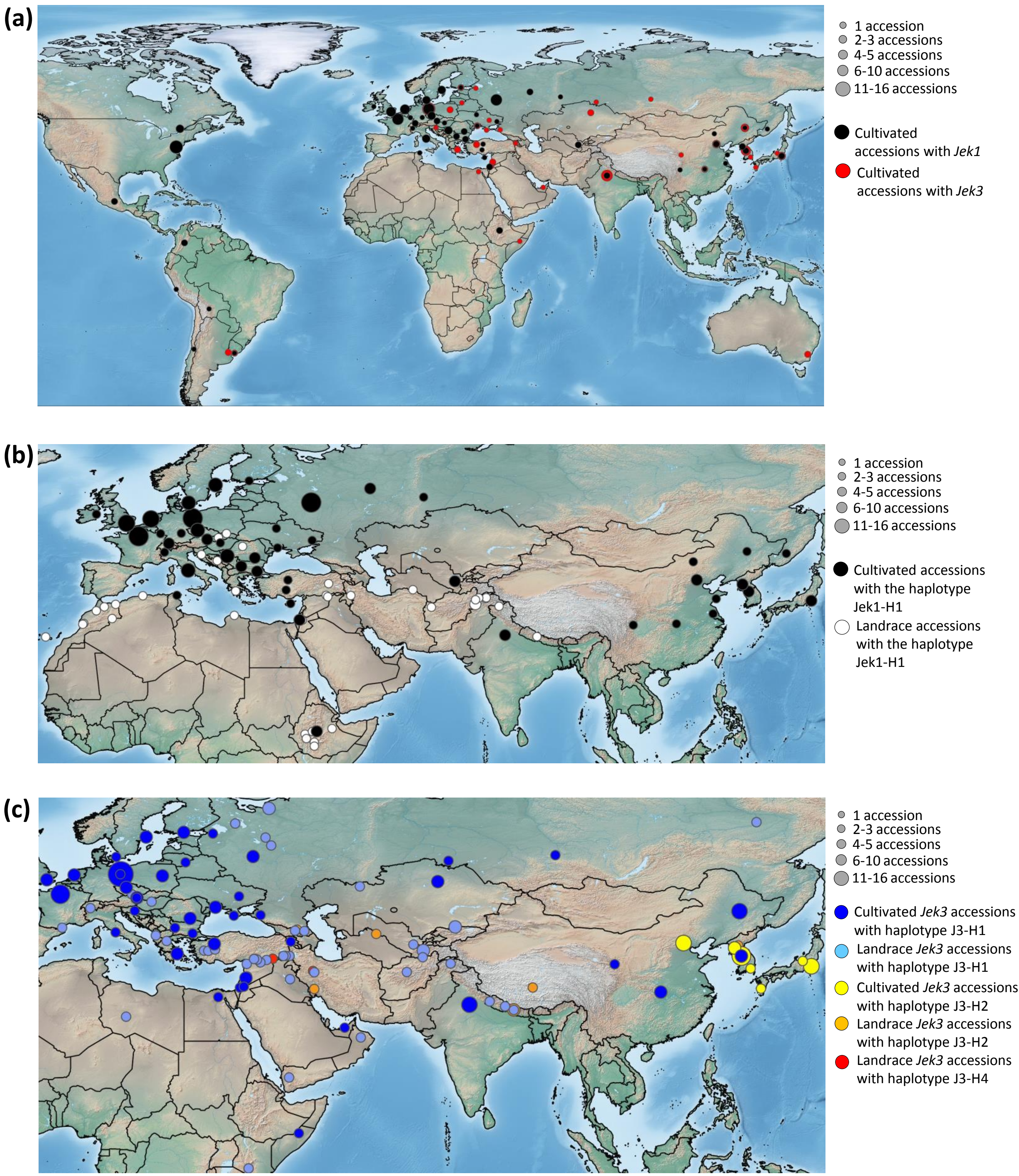


**Figure S6.** Geographical distribution of wild and cultivated barleys with *Jek1* and *Jek3*. (a) Geographical distribution of cultivated accessions with *Jek1* comprising single J1-H1 haplotype and *Jek3* comprising two haplotypes J3-H1 and J3-H2. (b) Geographical distribution of landraces and cultivars bearing J1-H1 haplotype. (c) Geographical distribution of landraces and cultivars bearing domesticated *Jek3* haplotypes.
