# Supplementary material for: The highly divergent Jekyll genes, required for sexual reproduction, are lineage specific for the related grass tribes Triticeae and Bromeae
Source: Plant J. 2019 May 25;98(6):961–74. doi: 10.1111/tpj.14363 (PMC6851964; doi:10.1111/tpj.14363)
Supplement: Supplementary file 7 — Figure S7. Phylogenetic tree of selected species from the Poaceae family used in the present study and in Radchuk et al. (2006). [file TPJ-98-961-s007.pdf]

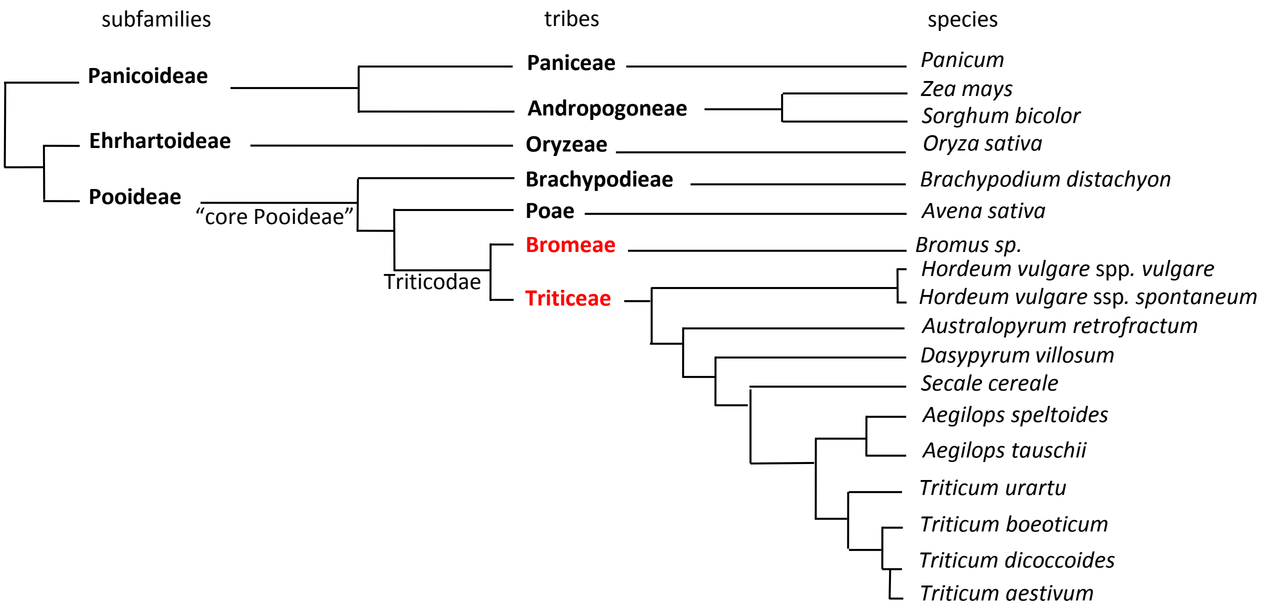

Figure S7. Phylogenetic tree of selected species from the Poaceae family used in the present study and in Radchuk et al. (2006). The tribes with Jek genes are shown in red. The phylogenetic distances are not to scale.
