# Supplementary material for: The highly divergent Jekyll genes, required for sexual reproduction, are lineage specific for the related grass tribes Triticeae and Bromeae
Source: Plant J. 2019 May 25;98(6):961–74. doi: 10.1111/tpj.14363 (PMC6851964; doi:10.1111/tpj.14363)
Supplement: Supplementary file 8 — Figure S8. Distribution of Jek sequences in Aegilops speltoides population. [file TPJ-98-961-s008.pdf]

(a)

```
Ae J1-H1 MAARGGKALVLAMLISFLAVQGTLGDLHKCFCGCYTKCMKQTAGHDACVKQCMNPHGKCFFGCRRKSTPSMMALADHVNPDRDEDTMTVAAEGADHGKFNVTEGSMAVPAGGADHGEFNVTEGSMAALAGGADHGEFNVTEEDAAP  147
Hv J1-H2 ............................................................................................................................................A......  147
```

(b)

```
Ae J3-H1 MAARGGKALVLAMLISFLAVQGTLGLDFAACYCQHNKWCMKLMPPGSGTELYCLKKSIRECTSLRCSKRAPSVMPLADPVNPVTDEGSMAVPAGGADHGEFHITE-------------------GSVAAAAGGADHGDFNTAEEDAAP 130
Ae J3-H2 ......................................................................................................V..-------------------.............E..V....... 130
Ae J3-H3 ..................V......F..V....G.HSA..IHT.RS..Q.FH.M.T.LAA..GW.....T.P......S..........................-------------------........................ 130
Ae J3-H4 .........................................................................................................-------------------........................ 130
Ae J3-H5 .............................................R...........................................................-------------------........................ 130
Hv J3-H1 .......................PVF.......HLH.G.LLHT.AS.TK.FH.VMT.LAT..DR....IT........A..--..................NVA.RSMAAPAEGADHSQFNFAE.NM..P....H..ES......... 147
```

(c)

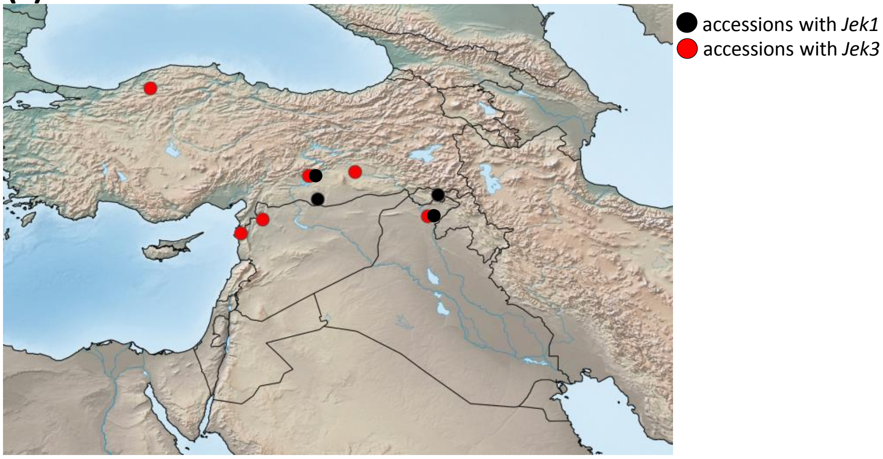

Figure S8. Distribution of *Jek* sequences in *Aegilops speltoides* population. (a, b) comparison of deduced amino acid sequences for Jek1 (a) and Jek3 (b) haplotypes of *Ae. speltoides* (Ae) with the corresponding proteins from barley (Hv). The amino acids identical to the Ae J1-H1 haplotype in (a) and to Ae J3-H1 haplotype in (b) are hidden in the other haplotypes. Wild barley J1-H2 haplotype is used in a comparison. (c) Geographical distribution of *Jek1* and *Jek3* variants in *Ae. speltoides* population.
