# Supplementary material for: The highly divergent Jekyll genes, required for sexual reproduction, are lineage specific for the related grass tribes Triticeae and Bromeae
Source: Plant J. 2019 May 25;98(6):961–74. doi: 10.1111/tpj.14363 (PMC6851964; doi:10.1111/tpj.14363)
Supplement: Supplementary file 9 — Figure S9. NMR imaging of lipid deposition in grains of selected wild Triticum species (a), two cultivars of domesticated T. aestivum (b) and five cultivars of Hordeum vulgare (c). [file TPJ-98-961-s009.pdf]

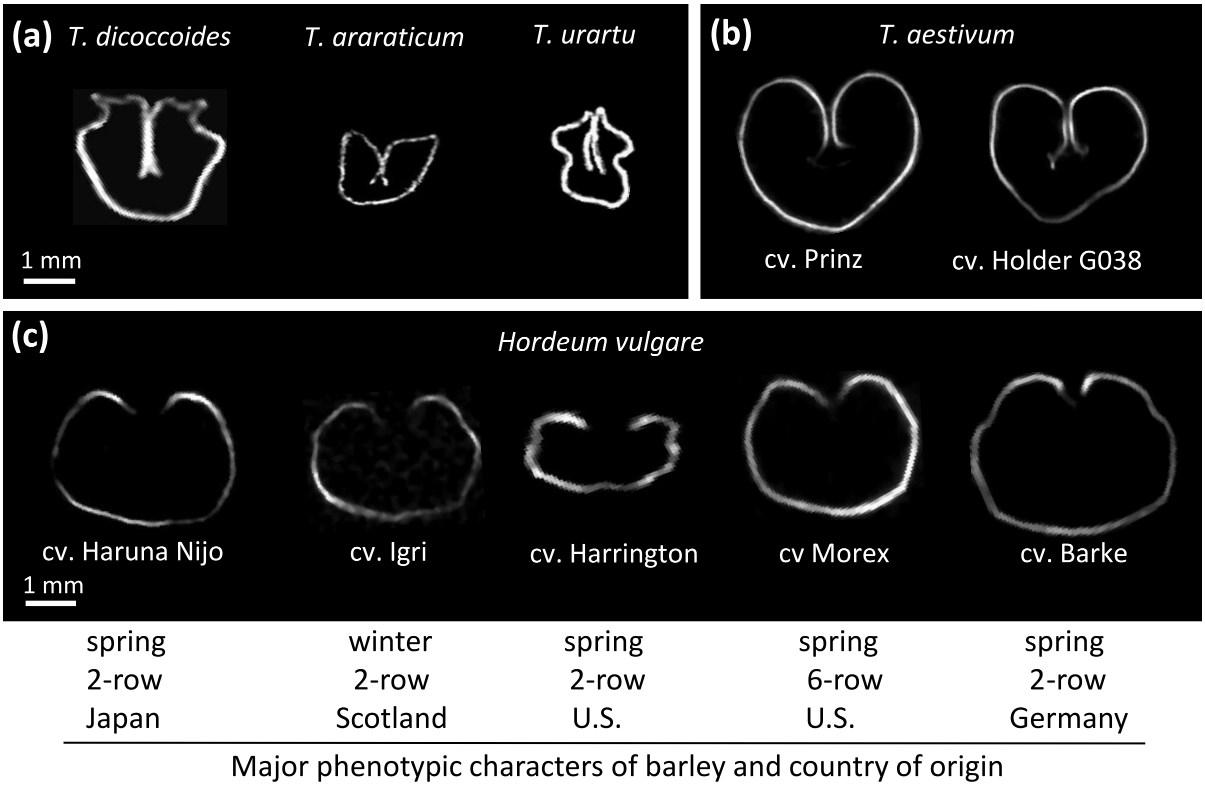

Figure S9. NMR imaging of lipid deposition in grains of selected wild *Triticum* species (a), two cultivars of domesticated *T. aestivum* (b) and five cultivars of *Hordeum vulgare* (c).
