# Supplementary material for: The highly divergent Jekyll genes, required for sexual reproduction, are lineage specific for the related grass tribes Triticeae and Bromeae
Source: Plant J. 2019 May 25;98(6):961–74. doi: 10.1111/tpj.14363 (PMC6851964; doi:10.1111/tpj.14363)
Supplement: Supplementary file 10 — Table S1. LOD scores and the proportion of observed phenotypic variation (R2) for the grain yield QTL assigned to the Jek locus on chromosome 3H in the Harrington × Morex and Steptoe × Morex mapping populations. [file TPJ-98-961-s010.docx]

**Table S1**. LOD scores and the proportion of observed phenotypic variation (R^2^) for the grain yield QTL assigned to the *Jekyll* locus on chromosome 3H in the Harrington *x* Morex and Steptoe *x* Morex mapping populations. SMA, single marker analysis; CIM, composite interval mapping analyses.

| Environment | LOD score, SMA | LOD score, CIM | R^2^ |
| --- | --- | --- | --- |
| Harrington *x* Morex population | | | |
| yieldMB95  yieldOR96  yieldSK96 | 2.8  2.5  4.7 | 4.8  4.0  8.7 | 0.10  0.10  0.15 |
| Steptoe *x* Morex population | | | |
| yld9  yld10  yld12  yld14  yld15  yld16 | 4.9  4.4  4.5  11.1  6.4  7.5 | 4.4  3.0  7.0  7.2  5.3  5.2 | 0.12  0.09  0.20  0.17  0.15  0.14 |
