# Supplementary material for: The highly divergent Jekyll genes, required for sexual reproduction, are lineage specific for the related grass tribes Triticeae and Bromeae
Source: Plant J. 2019 May 25;98(6):961–74. doi: 10.1111/tpj.14363 (PMC6851964; doi:10.1111/tpj.14363)
Supplement: Supplementary file 16 [file TPJ-98-961-s016.docx]

**Supporting Information**

**Figure S1.** Genomic structure of *Jek* genes. (a) Comparison of genomic fragments containing *Jek1* from Golden Gromise (GP) (contig­_81467) and *Jek3* from Morex genotype (contig_42242). (b) Comparison of *Jek2* genomic fragments derived from GP and Morex genotypes. Grey scale (below) represents the percentage of identity. 3’UTR, 3’ untranslated region; ATG, translation start; signal, signal peptide; stop, translation stop.

**Figure S2**. Inheritance and location of Jek1 and Jek3 sequences. (a) Inheritance of *Jek1* and *Jek3* sequences in F1 hybrids of Barke *x* Morex cross. (b) Segregation of *Jek1* and *Jek3* sequences in F2 plants of Barke *x* Morex cross. (c) Location of *Jek3* gene at 3H chromosome on a Steptoe *x* Morex (both contain Jek3) genetic map.

**Figure S3.** Co-location of *Jek1* and *Jek3* positions, and QTLs for grain yield on 3H chromosome as detected in two barley mapping populations. (a) Likelihood ratios (LR) for the grain yield QTLs revealed for Harrington *x* Morex cross in three environments. (b) Segregating *Jek1* and *Jek3* sequences among progenies of Harrington *x* Morex population as revealed by PCR amplification with gene-specific primers. (c) LR for the grain yield QTLs revealed for Steptoe *x* Morex cross in six environments. (d) CAPS marker developed to localize the *Jek1* sequence on the Steptoe *x* Morex genetic map. M, molecular marker; 1 and 2, amplified *Jek3* fragments from Morex (1) and Steptoe (2) correspondingly; 3 and 4, amplified *Jek3* fragments digested with Sac I enzyme from Morex (3) and Steptoe (4) correspondingly.

**Figure S4**. Expression profiles of *Jek1* and *Jek2* genes in the different tissues micro-dissected from the developing barley grains of cv. Barke.

**Figure S5.** Nucleotide alignments of haplotype sequences of *Jek1* (a) and *Jek3* (b). Protein-encoding sequences are shown in bolt, ATG start and stop codons are written in red. Sequences encoding putative peptide signals are labeled by red boxes, introns are labelled by black boxes.

**Figure S6.** Geographical distribution of wild and cultivated barleys with *Jek1* and *Jek3*. (a) Geographical distribution of cultivated accessions with *Jek1* comprising single J1-H1 haplotype and *Jek3* comprising two haplotypes J3-H1 and J3-H2. (b) Geographical distribution of landraces and cultivars bearing J1-H1 haplotype. (c) Geographical distribution of landraces and cultivars bearing domesticated *Jek3* haplotypes.

**Figure S7.** Phylogenetic tree of selected species from the Poaceae family used in the present study and in Radchuk et al. (2006). The tribes with *Jek* genes are shown in red. The phylogenetic distances are not to scale.

**Figure S8.** Distribution of *Jek* sequences in *Aegilops speltoides* population. (a, b) comparison of deduced amino acid sequences for Jek1 (a) and Jek3 (b) haplotypes of *Ae. speltoides* (Ae) with the corresponding proteins from barley (Hv). The amino acids identical to the Ae J1-H1 haplotype in (a) and to Ae J3-H1 haplotype in (b) are hidden in the other haplotypes. Wild barley J1-H2 haplotype is used in a comparison. (c) Geographical distribution of *Jek1* and *Jek3* variants in Ae. speltoides population.

**Figure S9.** NMR imaging of lipid deposition in grains of selected wild *Triticum* species (a), two cultivars of domesticated *T. aestivum* (b) and five cultivars of *Hordeum vulgare* (c).

**Table S1**. LOD scores and the proportion of observed phenotypic variation (R2) for the grain yield QTL assigned to the *Jekyll* locus on chromosome 3H in the Harrington *x* Morex and Steptoe *x* Morex mapping populations. SMA, single marker analysis; CIM, composite interval mapping analyses.

**Table S2.** List of barley accessions used in nucleotide diversity study.

**Table S3**. Population statistics of re-sequenced *Jek1*/*Jek3* accessions.

**Table S4.** *Jek1*/*Jek3*, *HvCEN*, *btr1*/*btr2* and *Ppd-H1* allelic combinations in domesticated barleys.

**Table S5**. List of primers used.

**Movie S1**. Comparative *in vivo* visualization of lipid distribution in barley (left) versus rice grains (right) by MRI. Lipid layer is absent in the region corresponding to the nucellar projection in the mature barley grain but present in the rice grain.
